# Structure-Based Discovery of *N*-Sulfonylpiperidine-3-Carboxamides as Novel Capsid Assembly Modulators for Potent Inhibition of HBV Replication

**DOI:** 10.3390/v14020348

**Published:** 2022-02-08

**Authors:** Yang Yang, Yu Yan, Jiaxin Yin, Jie Hu, Xuefei Cai, Jieli Hu, Jie Xia, Kai Wang, Ni Tang, Luyi Huang

**Affiliations:** Key Laboratory of Molecular Biology for Infectious Diseases (Ministry of Education), Department of Infectious Diseases, Institute for Viral Hepatitis, The Second Affiliated Hospital of Chongqing Medical University, Chongqing 400010, China; yangyangxd95@163.com (Y.Y.); yan199x@163.com (Y.Y.); YJX1134@163.com (J.Y.); biohujie@163.com (J.H.); 100052@cqmu.edu.cn (X.C.); hujieli1977@163.com (J.H.); 102773@cqmu.edu.cn (J.X.); wangkai@cqmu.edu.cn (K.W.)

**Keywords:** hepatitis B virus, core protein, capsid, assembly modulator, antiviral activity

## Abstract

As a key element during HBV replication, a nucleocapsid is considered a promising target for the treatment of chronic hepatitis B. The present study aimed to identify small molecules as novel capsid assembly modulators with antiviral activity. Structure-based virtual screening of an integrated compound library led to the identification of several types of HBV inhibitors. Among these inhibitors, *N*-sulfonylpiperidine-3-carboxamides (SPCs) potently reduced the amount of secreted HBV DNA. Through structure–activity relationship studies, we identified an SPC derivative, namely, C-39, which exhibited the highest antiviral activity without causing cytotoxicity. Mechanism studies showed that C-39 dose-dependently inhibited the formation of HBV capsid, synthesis of cccDNA, e antigen (HBeAg), viral pregenomic RNA (pgRNA), and HBV DNA levels, thereby restraining HBV replication. In summary, SPCs represent a new class of capsid assembly modulators. Further optimization of SPCs is expected to obtain new antiviral drugs against HBV infection.

## 1. Introduction

Chronic hepatitis B virus (HBV) was estimated to infect ~292 million people worldwide and causes chronic hepatitis, cirrhosis, and hepatocellular carcinoma (HCC) [1]. The World Health Organization estimated that approximately 887,000 people die from the resulting liver cirrhosis and hepatocellular carcinoma annually [2].

Currently, the treatment of chronic HBV infection is mainly based on nucleos(t)ide analogs (NAs) and pegylated interferon-alpha (peg-IFN-α) [3,4]. The response rates (HBsAg seroclearance) with NAs or peg-IFN-α treatment are low: ≤3% for NAs and 3–7% for peg-IFN-α [5]. The emergence of viral resistance, side effects, and virologic breakthrough after therapy discontinuation pose a major concern. As current treatments are limited, novel therapeutic approaches are needed. Agents with novel mechanism of action (MOA), alone or in combination, may increase cure rates and shorten treatment duration.

Capsid assembly is a pivotal step in the HBV life cycle and has been explored as a target for direct-acting antiviral agents [6]. One of the seven proteins encoded by HBV DNA is a core protein or capsid protein (Cp), which self-assembles to form a viral nucleocapsid. Cp plays a role in most steps of the well-described HBV life cycle [7,8]. Core proteins interact with the DNA polymerase, which binds to the epsilon (ε) sequence within the 5′ portion of pgRNA, thereby initiating nucleocapsid assembly [9]. Within the nucleocapsid, reverse transcription of the pgRNA by the polymerase produces relaxed circular DNA (rcDNA), single-stranded DNA (ssDNA), and double-stranded linear DNA (dslDNA). The rcDNA-containing capsids are either recycled back to the nucleus to replenish covalently closed circular DNA (cccDNA) or enveloped to form infectious viral particles [10,11]. Apart from playing an important role in viral DNA synthesis, Cp is also reported to be involved in the epigenetic regulation of cccDNA [12] and is responsible for the very early inhibition of the interferon response to HBV [13]. 

The capsid assembles with T = 3 or T = 4 icosahedral quasi-symmetry, which contains 90 or 120 core protein dimers, respectively. The T = 4 HBV particles are the most prevalent form and the infectious particles [14,15]. The full-length core protein consists of 183 amino acids and can be divided into the assembly domain (residues 1−149, also known as Cp149) and the nucleic-acid-binding domain (residues 150−183) [16]. Cp exists as a homodimer, which is the building block of a capsid. The assembly of a capsid is driven by the weak hydrophobic interaction (ranging from −2.9 to −4.4 kcal/mol) between the Cp dimer–dimer interfaces [17]. 

The weak interaction between Cp dimers also provides opportunities for regulation of the capsid assembly process by small molecules. Capsid assembly modulators (CAMs) bind to the pocket between Cp dimer–dimer interfaces, stabilize the complex, accelerate capsid assembly kinetics, and prevent encapsidation of the polymerase–pgRNA complex, thereby blocking viral DNA synthesis [18]. To date, multiple CAMs have been developed [19]. Heteroaryldihydropyrimidines (HAPs) [20,21] and dibenzothiazepines (DBTs) [22] misdirect Cp dimers to assemble aberrant noncapsid structures. Phenylpropenamides (PPAs) [23], sulfamoylbenzamides (SBAs) [24], phthalazinones [25], sulfamoylpyrrolamides (SPAs) [26], glyoxamoylpyrroloxamides (GLPs) [27], and pyrazolopyridinylsulfonamides (PPSs) [28] induce Cp dimers to assemble empty, morphologically intact capsids devoid of pgRNA and DNA polymerase.

**Figure 1:**
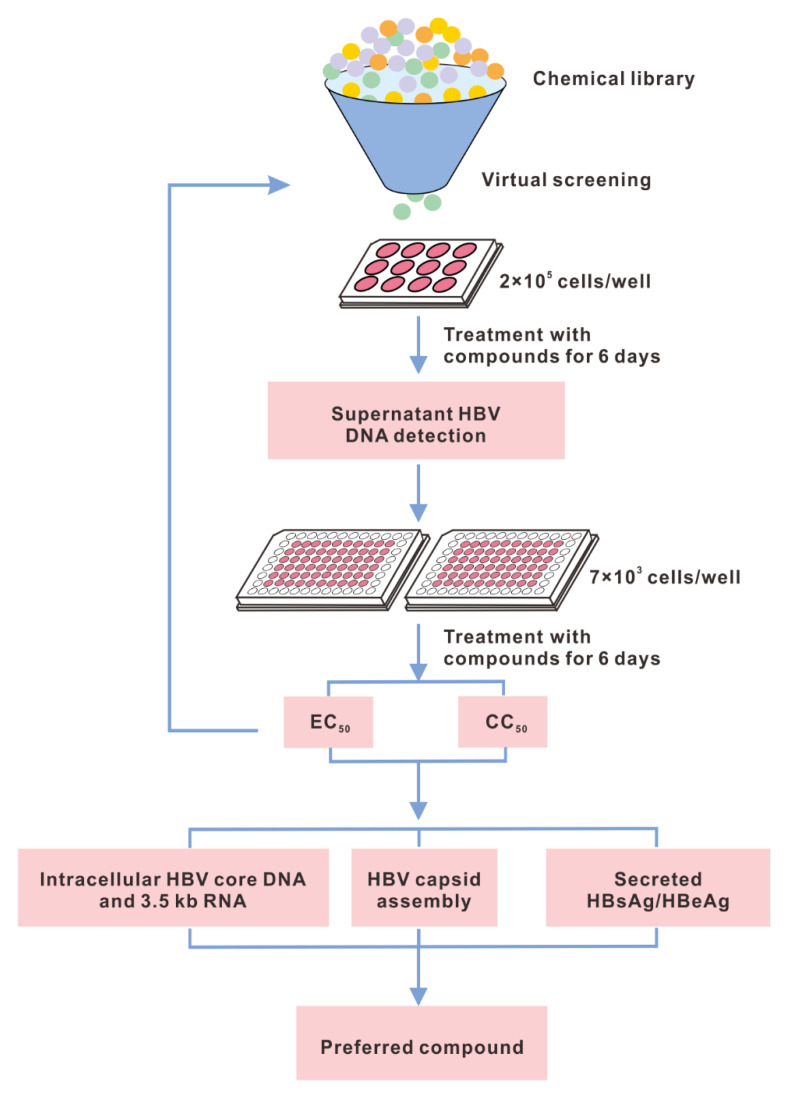
Schematic depiction of the primary screening workflow. HepAD38 or HepG2-HBV1.3 cells were treated with compounds (20 μM) for 6 days, then viral nucleic acids and proteins were detected.

To identify novel chemotypes of CAMs as leads for anti-HBV agent development, we screened potential compounds from a chemical library for their ability to inhibit the amount of supernatant HBV DNA secreted from HepAD38 cells, a stable cell line supporting high levels of HBV DNA replication. Cycles of screening identified a series of *N*-sulfonylpiperidine-3-carboxamide (SPC) derivatives as HBV inhibitors. Extensive biological assays, such as a capsid assembly assay, and quantification of the viral nucleic acids and proteins were employed to evaluate the preferred compound (Figure 1).

## 2. Materials and Methods

### 2.1. Compounds and Reagents

Entecavir (ETV) and BAY 41-4109 were purchased from TargetMol (Wellesley Hills, MA, USA). Compounds for screening were obtained from Chemdiv Inc. (San Diego, CA, USA) and Vitas M Labs (Champaign, IL, USA). The ^1^H NMR spectra of selected compounds are provided in the Supplementary Materials.

### 2.2. Cell Culture

Human HCC cell lines HepG2 were obtained from the American Type Culture Collection (HB-8065, ATCC, Manassas, VA, USA); and HepAD38 cells, which express pre-genomic RNA (pgRNA) under the control of the inducible tetracycline promoter, were kindly provided by Prof. C. Seeger (Institute for Cancer Research, Fox Chase Cancer Center, Philadelphia, PA, USA). HepG2 and HepAD38 cells were maintained in Dulbecco’s modified Eagle’s medium (DMEM, HyClone, Logan, UT, USA) supplemented with 10% fetal bovine serum (FBS, Gibco, Rockville, MD, USA), 100 units/mL penicillin, and 100 μg/mL streptomycin (HyClone). HepAD38 cells were cultured in the presence of another 500 μg/mL G418 (Sangon Biotech, Shanghai, China) to maintain the stably transfected HBV genome, and 1 μg/mL tetracycline (Sangon Biotech) was added to suppress HBV transcription. HepG2 cells were infected with adenovirus Ad-HBV1.3, provided by Prof. Michael Nassal (University Hospital Freiburg, Freiburg, Germany), for 12 h to sustain all processes of HBV replication.

### 2.3. Infection of HepG2-NTCP with HBV Particles

HepG2 cell stably expressing sodium taurocholate co-transporting polypeptide (HepG2-NTCP) was a gift from Ningshao Xia (Xia Men University, Xiamen, China) and was infected with HBV virus as previously described [29]. Briefly, HepG2-NTCP cells were cultured in 12-well plates with the Williams’ E media for 12 h, after which, the cells were infected with HBV particles (the MOI was 1000 genome equivalents per cell) and simultaneously treated with the indicated compound(s) for another 24 h. After removing the virus-containing medium, the cells were cultured in the presence of the indicated compound(s) for another 7 days.

### 2.4. Cytotoxicity Assay

A Cell Counting Kit-8 (CCK-8, Topscience, Shanghai, China) was used to detect compound cytotoxicity according to the manufacturer’s instructions. Briefly, approximately 7 × 10^3^ cells were seeded in 96-well culture plates and treated with compounds or DMSO for 6 days after cell adherence. The culture medium was replaced with fresh media containing compounds at 72 h intervals. After the 6-day treatment, cells were incubated with 100 μL medium containing 10% CCK-8 for approximately 30 min at 37 °C. Then, optical density (OD) values were determined at 450 nm using a microplate reader. 

### 2.5. Quantification of Secreted and Intracellular HBV DNA

Cells were treated with indicated compounds for 6 days as previously described [28]. For secreted HBV DNA, HepAD38 and HepG2-HBV1.3 media were extracted via a boiling method according to the manufacturer’s instructions (Kehua Bio-Engineering, Shanghai, China). Briefly, the cell supernatant was mixed with solution A (mainly containing NaCl) and centrifuged at 13,000× *g*; then, the supernatant was removed. Next, solution B (containing Tris and NP-40) was added to the tube. After vortexing and a short spin, the mixture was subject to a boiling water bath at 100 °C for 10 min and then centrifuged for 10 min at 13,000× *g*. The supernatant was used to quantify secreted HBV DNA via RT-qPCR.

For intracellular HBV core DNA, cells were collected and lysed at 37 °C for 30 min with a cell lysis buffer (10 mM Tris-HCl (pH 8.0), 1 mM EDTA, 2% sucrose, and 1% NP-40). The mixture was centrifuged at 13,000× *g* for 5 min. Thereafter the supernatant was treated with micrococcal nuclease (70196Y, Affymetrix, Santa Clara, CA, USA) and 10 mM CaCl_2_ for 60 min at 37 °C to destroy non-packaged nucleic acid (or remove all noncore protected nucleic acid). A final concentration of 10 mM EDTA was used to terminate the reaction. After precipitation with 35% polyethylene glycol (PEG) 8000, digestion with 0.5 mg/mL proteinase K (3115879001, Roche Diagnostics GmbH, Mannheim, Germany) took place at 45 °C for 12 h. Nucleic acids were extracted using phenol/chloroform/isoamylol (25:24:1, Solarbio, Beijing, China) three times and precipitated with ethanol. The isolated HBV DNA was subjected to RT-qPCR using SYBR Green qPCR Master Mix (Bio-Rad, Hercules, CA, USA). The plasmid pCH9/3091 (containing 1.1 copies of HBV genome) served as a template for the standard curve. The primers are listed in Table S1. 

### 2.6. HBV cccDNA Isolation and Taq-Man Probe RT-qPCR

HepG2-NTCP cells were infected with HBV and treated with different compounds, either together with the viral inoculum (day 0) or at 4 days after infection (day 4). cccDNA in cultured cells were isolated using a modified Hirt method after infection for 8 days. Briefly, cells were lysed in 500 μL SDS lysis buffer (50 mM Tris-HCl (pH 8.0) and 10 mM EDTA, 150 mM NaCl, 1% SDS) at 37 °C for 20 min. Then, 100 μL 5 M NaCl was added into the cell lysate and incubated at 4 °C overnight. After centrifugation at 12,000× *g* for 30 min, the supernatant was collected. Following purification with phenol/chloroform (24:1, Solarbio), the DNA was precipitated with ethanol and finally dissolved in a TE buffer. All samples were treated with plasmid-safe adenosine triphosphate (ATP)-dependent deoxyribonuclease (PSAD) (Epicentre, Lucigen Corporation, Middleton, WI, USA) at 37 °C for 12 h, followed by incubation at 70 °C for 30 min to inactivate PSAD. For HBV cccDNA amplification, we used TaqMan primers (listed in Table S1) to specifically amplify cccDNA.

### 2.7. Quantification of Intracellular HBV RNAs and Northern Blotting

Total RNAs were extracted with TRIzol reagent (Invitrogen, Carlsbad, CA, USA), and reverse transcribed using PrimeScript™ RT Reagent Kit with gDNA Eraser (RR047A, TaKaRa, Tokyo, Japan) following the manufacturer’s instructions. RT-qPCR was performed to quantify mRNA levels using the SYBR Green qPCR Master Mix. HBV 3.5 kb RNA was standardized to β-actin. Primers are listed in Table S1. 

Encapsidated pgRNA was purified as previously described [30,31]. Briefly, cells were lysed in 200 μL of lysis buffer (50 mM Tris-HCl (pH 7.5), 1 mM EDTA, 150 mM NaCl, 1% NP-40) and the nuclei were removed via centrifugation. All the samples were incubated with micrococcal nuclease and CaCl_2_ for 1 h at 37 °C to digest free nucleic acids. The reaction was stopped with 0.5 M EDTA, then encapsidated pgRNA was extracted with TRIzol -LS reagent (Invitrogen, Carlsbad, CA, USA). Total RNAs and encapsidated pgRNA were electrophoresed in 1% agarose gel, transferred onto a nylon membrane, and immobilized by UV cross-linking. Hybridization was performed at 68 °C overnight in a buffer containing 100 ng DIG-UTP labeled minus strand HBV riboprobe. The DIG Northern Starter Kit (12039672910, Roche Diagnostics GmbH, Mannheim, Germany) was used to detect HBV RNAs. 

### 2.8. Southern Blotting

Southern blotting was performed as previously described [29]. Extracted DNA samples were separated via electrophoresis in 1.2% agarose gel and transferred onto a nylon membrane (11417240001, Roche Diagnostics GmbH, Mannheim, Germany). After being soaked in denaturation solution (0.5 M NaOH and 1.5 M NaCl) and then neutralized in 1 M Tris-HCl (pH 7.4) and 1.5 M NaCl, the membrane was fixed via UV cross-linking. Then, a digoxigenin (DIG)-labeled full-length HBV genome probe (DIG high prime DNA labeling and a detection starter kit, Roche Diagnostics GmbH, Mannheim, Germany) was used to detect the HBV DNA via hybridization.

### 2.9. Detection of HBV Antigens

Quantification HBsAg and HBeAg in culture supernatants were assayed via enzyme-linked immunosorbent assay (ELISA) kits (Kehua Bio-Engineering, Shanghai, China), according to the manufacturer’s protocols. ELISA results are presented as 100% × S/VC (S = sample OD value and VC = vehicle control OD value).

### 2.10. Immunofluorescence

HepAD38 cells were treated with BAY 41-4109, C-18, or C-39 for 3 days, and then seeded on cover glasses in 12-well plates. After being fixed with 4% paraformaldehyde for 25 min at room temperature, the cells were incubated with a primary antibody (anti-HBc, 1:1000, B0586, Dako) overnight. Then, the cells were washed in PBS and incubated with Alexa Fluor^®^ 488 dye conjugated anti-rabbit antibody (MA-1-91878, Thermo, Waltham, MA, USA). Finally, cells were mounted with 4′,6′-diamidino-2-phenylindole (DAPI) and images were taken using a Leica confocal microscope.

### 2.11. Drug Susceptibility Assay

A recombinant plasmid pNeo-CH9/3091 containing HBV 1.1 ploidy and CMV promoter (HBV1.1-WT) was used as a template to construct the ETV-resistant mutant (rtL180M/S202G/M204V) plasmid via site-directed mutagenesis [32,33]. HepG2 cells were transfected with an HBV1.1-WT or ETV-resistant mutant (rtL180M/S202G/M204V) construct. Then, the cells were treated with increasing concentrations of ETV or C-39 for 6 days after a 36 h transfection. After the treatment, secreted HBV DNA was detected, as described above.

### 2.12. Analysis of Intracellular HBV Capsid

For detecting HBV capsids, experiments were performed as previously described [34,35,36,37]. A total of 2 × 10^5^ HepAD38 or HepG2-HBV1.3 cells were seeded in 12-well plates. After 6–8 h, the cells were incubated with the indicated compounds for 6 days, with a change of compounds containing fresh medium on the third day. Cells were then lysed with lysis buffer (containing 1% NP40) for 10 min, and cell debris and nuclei were removed via centrifugation at 13,000× *g*. Then, samples were separated on 1.2% non-denaturing agarose gel electrophoresis and HBV capsids were transferred from the gel onto a nitrocellulose membrane via capillary transfer in a TNE buffer (pH 7.4, 10 mM Tris-HCl, 150 mM NaCl, and 1 mM EDTA) overnight. On the second day, the membrane was fixed with 50% methanol and HBV capsids were detected with anti-HBV core antibody (1:1000, B0586, Dako).

### 2.13. Purification of Cp149 and Transmission Electron Microscopy

The Cp149 protein (amino acids 1-149, the assembly domain of the HBV core protein) was used in an electron microscopy study to determine the effect of the compound C-39 on HBV capsid assembly in vitro [38]. The protein was expressed in E. coli and purified as previously described [39,40]. The purified Cp149 (5 μM dimer) was assembled in reaction buffer (150 mM HEPES, 300 mM NaCl, pH 7.5) at a ratio of 2:1 in the presence and absence of C-39. The reaction mixture was incubated at room temperature for 3 and 7 days in vitro. After the assembly reaction, a 10 μL solution was negatively stained via incubating on a carbon-coated grid for 10 min; then, the grid was washed with water and stained with 3% uranyl acetate for 1 min. The results of the TEM were supported by Beijing Zhongkebaice Technology Service Co., Ltd. (Beijing, China). 

### 2.14. Virtual Screening and Molecular Modeling

The model was extracted from the crystal structure of HBV Cp (Y132A) and SBA_R01 (NVR 3-778) complex (PDB code: 5T2P). The macromolecule was prepared using AutoDockTools [41]. Small molecules from ChemDiv and Vitas-M libraries were prepared using Open Babel [42] and were docked into the SBA binding site using AutoDock Vina [43]. The results were presented using PyMOL (Schrödinger LLC, New York, NY, USA).

### 2.15. Statistical Analysis

Data are expressed as mean ± standard deviation (SD). Student’s *t*-test was used to assess the statistical significance between combination against any other treatment. Multiple comparisons were determined using a one-way analysis of variance. A value of *p* less than 0.05 was considered to indicate statistical significance.

## 3. Results

### 3.1. Virtual Screening and Antiviral Activity Measurement of Potential HBV CAMs

**Figure 2:**
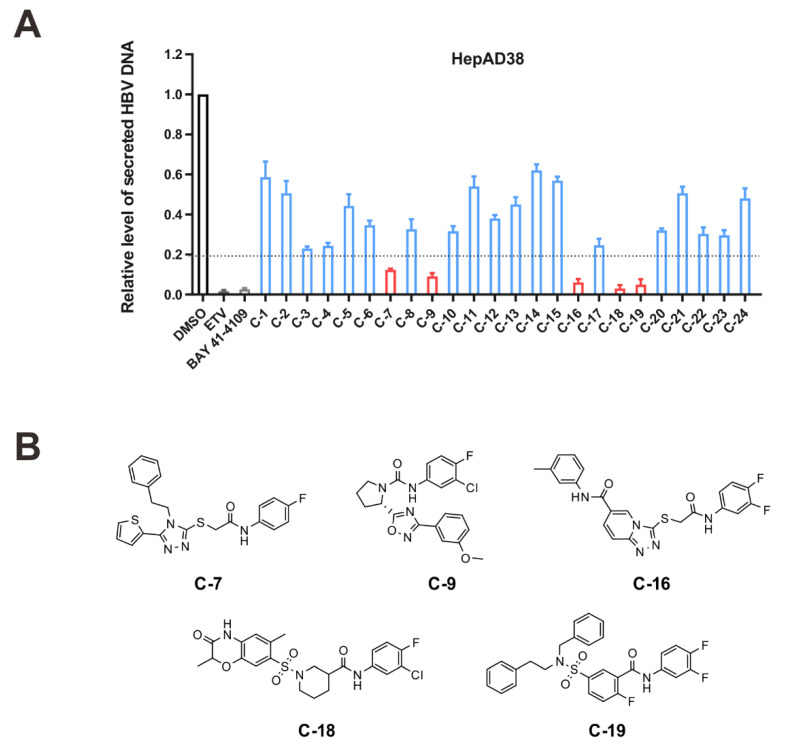
Screening and identification of novel HBV inhibitors. (**A**) Effects of the 24 compounds (C-1–C-24, 20 μM) on secreted HBV DNA in HepAD38 cells. Compounds with significant antiviral activity were defined with a threshold of 20% of DMSO control. (**B**) Structures of the initial hit compounds C-7, C-9, C-16, C-18, and C-19. Data are representative of three independent experiments and are expressed as the mean ± SD.

To discover novel HBV capsid assembly modulators, we first screened commercial chemical libraries (about 3 million compounds) using a high-throughput docking campaign. After iterative cycles of modeling, 24 compounds in stock were consequently selected (Table S2). The compounds were tested for their ability to suppress HBV DNA levels in culture supernatants of a stably transfected HBV cell line HepAD38. Impacts on the HBV DNA were also observed with 25 nM entecavir (ETV) and 2 μM BAY 41-4109, which served as positive controls. The results showed that five compounds (C-7, C-9, C-16, C-18, and C-19) reduced extracellular HBV DNA to less than 20% of the negative control (DMSO) (Figure 2A), corresponding to a hit rate of 21%. Apart from C-19, the other four compounds had novel scaffolds (Figure 2B). 

### 3.2. Anti-HBV Activity Verification and Molecular Interaction Analysis

**Figure 3:**
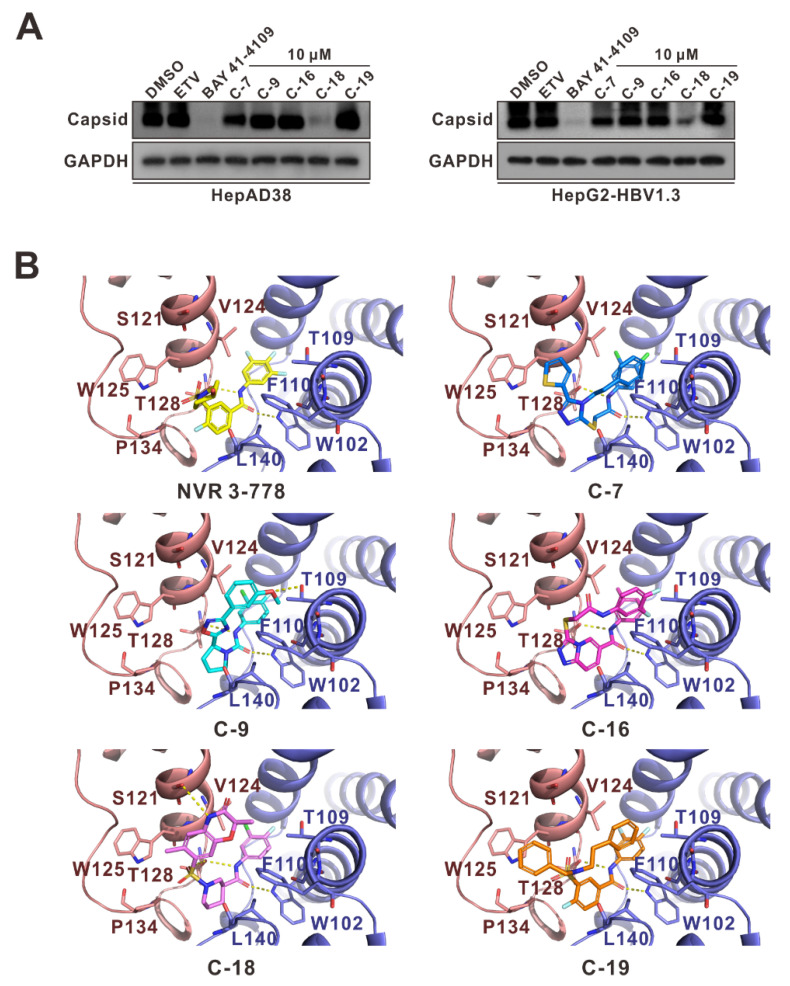
Effects of initial hits on viral particles and molecular interaction analysis. (**A**) Effects of five initial hits on intracellular HBV capsid formation. HepAD38 or HepG2-HBV1.3 cells were incubated with C-7, C-9, C-16, C-18, C-19 (10 μM), ETV (25 nM), or BAY 41-4109 (2 μM) for 6 days. HBV core particles were isolated from cell lysates by 1.2% non-denaturing agarose gel electrophoresis and immunoblotting. (**B**) Putative molecular interactions between HBV core protein dimers and NVR 3-778, C-7, C-9, C-16, C-18, or C-19. Compounds and residues were represented as sticks. Yellow dashed lines indicated the hydrogen bonds.

Next, we determined the EC_50_ and CC_50_ values of the five initial hits. As shown in Table 1, all the compounds inhibited the HBV DNA secretion within the low-micromolar-to-nanomolar range with a selectivity index (SI) of more than 20. Specifically, C-18, an SPC derivative, had the strongest inhibition effect (EC_50_ = 0.11 μM), but the cytotoxicity was the highest (CC_50_ = 14.8 μM). Moreover, only C-18 strongly inhibited HBV capsid formation at 10 μM. C-7 exhibited moderate inhibition and the other three compounds (C-9, C-16, and C-19) did not affect the capsid formation (Figure 3A).

After verification of the initial hits, we analyzed the intermolecular interactions between the HBV core proteins and the CAMs. With the complex structure of Cp and NVR 3-778 (PDB ID: 5T2P) as a template, we constructed artificial complexes of interest by molecular docking (Autodock Vina). The five compounds were docked into the cavity located in the interface between two Cp dimers (HAP/SBA binding site). Small molecules binding to this pocket were used to induce misassembly of the core protein or the formation of empty capsid [38]. All the compounds tethered two HBV core proteins by interacting with side chains of W102 and T128, and C-18 formed an additional hydrogen bond with S121 (Figure 3B), which could stabilize the complex and accelerate Cp assembly kinetics. 

### 3.3. C-18 Inhibited Capsid Formation and HBV Replication

**Figure 4:**
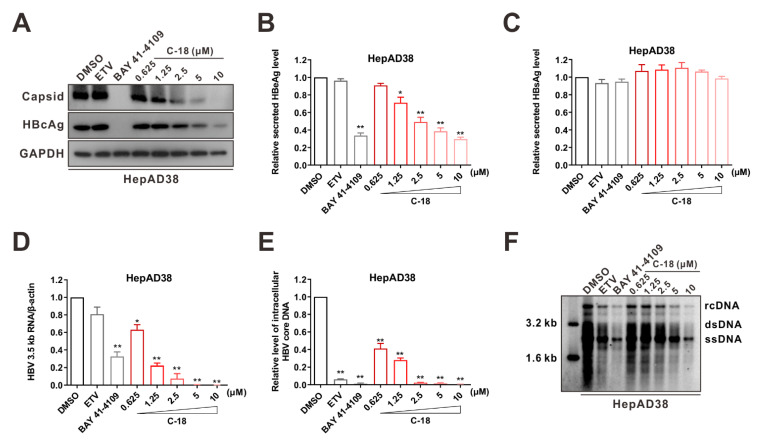
C-18 inhibits HBV replication in HepAD38 cells. (**A**) Effects of C-18 on intracellular HBV capsid formation and HBc. HepAD38 cells were incubated with the indicated concentrations of C-18 for 6 days. Capsids and HBV core protein were probed with anti-HBV core antibody, and GAPDH was used as the loading control. (**B**–**F**) Effects of C-18 on HBV replication. HepAD38 cells were incubated with the indicated concentrations of C-18 for 6 days. Secreted HBeAg (**B**) and HBsAg (**C**) were determined using ELISA, intracellular HBV 3.5 kb RNA (**D**) and HBV DNA (**E**) were measured using qPCR, and intracellular HBV replication intermediates (RIs) were detected using Southern blot analysis. rcDNA, relaxed circular DNA; dsDNA, double-stranded DNA; ssDNA, single-stranded DNA. (**F**) ETV (25 nM) and BAY 41-4109 (2 μM) were used as controls. The data in (**B**–**E**) are representative of three independent experiments and are expressed as mean ± SD (Student’s *t*-test was used to assess the statistical significance between DMSO group against any other treatment; * *p* < 0.05, ** p < 0.01).

To explore the effect of compound C-18 on HBV capsid formation and HBV replication, HepAD38 cells were treated with C-18 at concentrations ranging from 0.625 μM to 10 μM for 6 days. HBV capsid formation and the core protein were analyzed using agarose gel electrophoresis, the results indicated that C-18 inhibited intracellular HBV capsid formation and core protein in a dose-dependent manner (Figure 4A). Furthermore, C-18 reduced the HBeAg level in the culture supernatant (Figure 4B, *p* < 0.05). However, it had no impact on HBsAg (Figure 4C). In addition, treatment with C-18 led to a dramatic decrease of the HBV 3.5 kb RNA and intracellular HBV core DNA levels (Figure 4D,E, *p* < 0.01), as determined using quantitative polymerase chain reaction (qPCR). Southern blot indicated that HBV replicative intermediates were notably downregulated by C-18 (Figure 4F). Transient HBV-replicating cells (HepG2-HBV1.3) behaved similarly to HepAD38 cells in response to the C-18 treatment. The EC_50_, CC_50_, and SI were 0.14 μM, 20.6 μM, and 147.1, respectively. C-18 downregulated capsid formation, HBeAg secretion, intracellular 3.5 kb RNA, and core DNA in HepG2-HBV1.3 cells in a dose-dependent manner (Figure S1). Taken together, our data suggested that C-18 potently inhibited the HBV replication cycle.

### 3.4. Structure−Activity Relationship (SAR) Analysis of SPCs 

**Figure 5:**
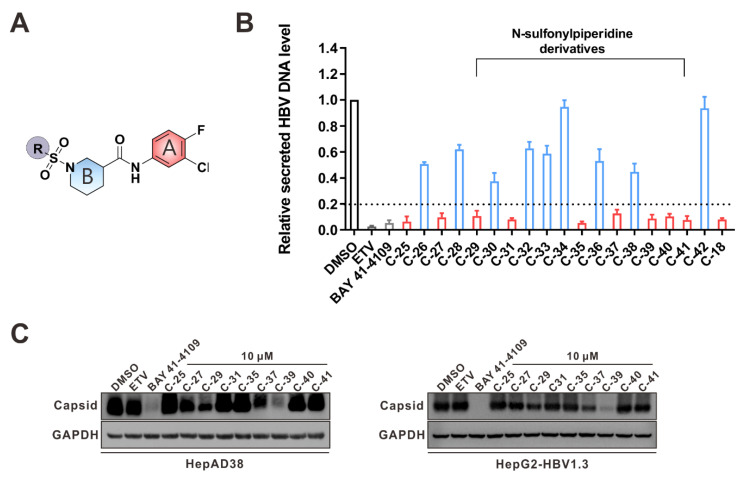
Effects of the rescreened compounds on HBV replication and capsid formation. (**A**) The scaffold of C-18. The substituent on the SPC scaffold was represented as an R group. Ring A and ring B are highlighted. (**B**) Effects of the 18 compounds (C-25–C-42) and C-18 on secreted HBV DNA in HepAD38 cells. The threshold for hit selection was set stringently to a 20% residual level relative to the control (DMSO). Data are representative of three independent experiments and are expressed as the mean ± SD. (**C**) Effects of nine selected compounds on intracellular HBV capsid formation. HepAD38 and HepG2-HBV1.3 were incubated with nine selected compounds (10 μM), ETV (25 nM), and BAY 41-4109 (2 μM) for 6 days.

C-18 is the compound that temporarily exhibits the highest anti-HBV activity. However, it is poisonous to the cells (HepAD38, CC_50_ = 14.8 μM; HepG2, CC_50_ = 20.6 μM). In order to improve its antiviral activity and reduce the cytotoxicity, we further explored the chemical space around its scaffold by introducing modifications at the aryl position (group R, Figure 5A). A total of eighteen compounds (C-25–C-42) were selected, where thirteen of them (C-29–C-41) bore substituents at the group R position on the SPC scaffold (Figure 5A, Table 2). Anti-HBV activity tests yielded nine compounds (C-25, C-27, C-29, C-31, C-35, C-37, C-39, C-40, and C-41) that drastically inhibited HBV DNA secretion (the residual HBV DNA level was less than 20% of DMSO group; Figure 5B).

Subsequent inhibition curves and cytotoxicity measurements indicated that all nine of these compounds effectively inhibited the secretion of HBV DNA, with EC_50_ values of 0.29 μM, 1.12 μM, 1.29 μM, 0.26 μM, 0.41 μM, 0.35 μM, 0.056 μM, 0.39 μM, and 0.062 μM, respectively (Table 2). The hit rate was much improved compared with the initial screening. Seven of thirteen SPCs inhibited the HBV DNA level to less than 20% of the control. C-39 and C-40 were the top two compounds with anti-HBV activities. C-39 efficiently inhibited HBV DNA in HepAD38 (EC_50_ = 0.056 μM, SI > 1786) and HepG2-HBV1.3 (EC_50_ = 0.075 μM, SI > 1333) cells. When the *N*-methyl group on pyrrole was removed, the activity of the corresponding compound (C-37) decreased. C-29, C-35, and C-40 still exhibited medium cytotoxicity, where only C-39 displayed no obvious toxicity with CC_50_ greater than 100 μM.

Then, we detected their effects on capsid formation. The results showed that in HepAD38 and HepG2-HBV1.3 cells, only C-39 and the known type I CAM BAY 41-4109 dramatically inhibited capsid formation, while ETV and the other eight compounds showed much less or no inhibition effects (Figure 5C). Taking into account both the potency and the action mechanism, C-39 is appropriate for the subsequent antiviral exploration.

### 3.5. C-39 Inhibited HBV Replication In Vitro

**Figure 6:**
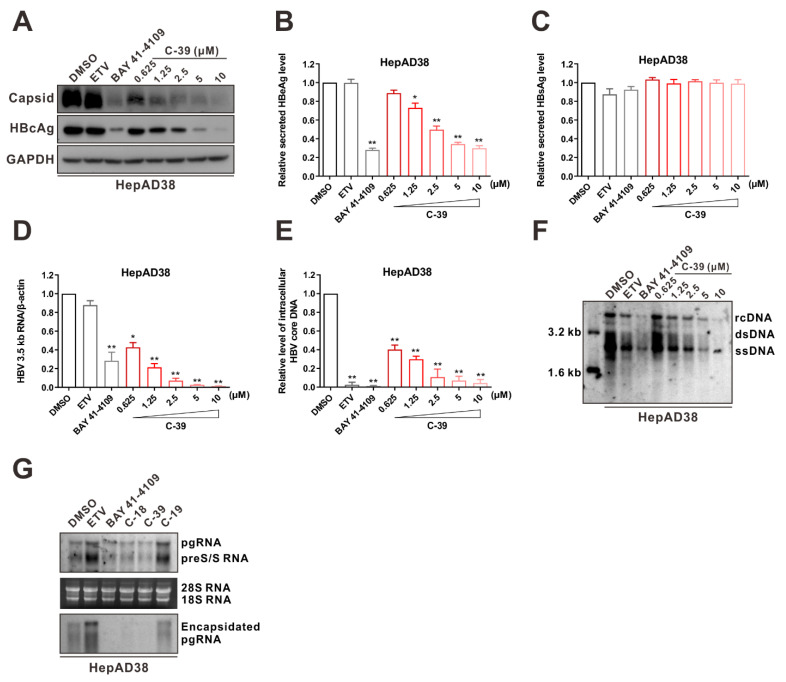
C-39 significantly inhibits HBV replication. HepAD38 cells were incubated with the indicated concentrations of C-39 for 6 days. (**A**) Effects of C-39 on intracellular HBV capsid formation. HBV capsids and core protein were assessed using the anti-HBV core antibody. (**B**–**F**) Effects of C-39 on HBV replication. Secreted HBeAg (**B**) and HBsAg (**C**) were determined using ELISA, intracellular HBV 3.5 kb RNA (**D**) and core DNA (**E**) were measured using qPCR, and intracellular HBV replication intermediates (RIs) were assessed using Southern blotting (**F**). (**G**) Effects of C-18, C-39, and C-19 (5 μM) on the HBV total RNAs and encapsidated pgRNA were determined using Northern blotting. ETV (25 nM) and BAY 41-4109 (2 μM) were used as controls. The data in (**B**–**E**) are representative of three independent experiments and are expressed as mean ± SD (Student’s *t*-test was used to assess the statistical significance between DMSO group against any other treatment; * *p* < 0.05, ** p < 0.01).

Next, the effects of C-39 on the HBV capsid, as well as the core protein in HepAD38 and HepG2-HBV1.3 cells, were assessed. As shown in Figure 6A and Figure S2A, C-39 inhibited the HBV capsid formation and downregulated the core protein in a concentration-dependent manner. We further investigated the anti-HBV activity of C-39 in HepAD38 and HepG2-HBV1.3 cells by quantifying HBeAg, HBsAg, HBV RNAs, and HBV DNA. In both cell lines, C-39 markedly reduced secreted HBeAg, intracellular 3.5 kb RNA, and HBV core DNA levels with high-nanomolar to low-micromolar potency (Figure 6B,D–F and Figure S2B,D–F). At the same time, C-39, ETV, and BAY 41-4109 had no impact on HBsAg production (Figure 6C and Figure S2C). We then examined the HBV encapsidated pgRNA level with the treatment of the capsid assembly disruptors C-18, C-39, and non-disruptor C-19 using Northern blot hybridization, as shown in Figure 6G. With the long-timescale treatment of BAY 41-4109, C-18, or C-39, HBV encapsidated pgRNA was markedly downregulated, along with a slight decrease in intracellular total HBV RNAs, whereas C-19 exhibited slight effects. In contrast, encapsidated pgRNA was upregulated upon ETV treatment.

HepG2-NTCP cells, which express NTCP in a doxycycline (Dox)-inducible manner, were infected by HBV virions prepared from HepAD38 cultures at an MOI of 1000 and simultaneously treated with DMSO or compounds. Similar to BAY 41-4109, C-18 and C-39 demonstrated inhibition of capsid formation, HBc, HBeAg, HBV 3.5 kb RNA, and intracellular core DNA production, except HBsAg (Figure S3A–E). They also prevented the formation of cccDNA during the early postentry step, whereas they had a minor effect on pre-established cccDNA formation (Figure S3F,G). Unsurprisingly, ETV only suppressed intracellular core DNA.

### 3.6. C-39 Remained Sensitive to Nucleos(t)ide-Resistant HBV Variant and Showed Additive Antiviral Activity In Vitro with ETV

New drugs or drug combinations that are active against nucleos(t)ide-resistant virus variants could provide a treatment option for patients with drug-resistant HBV. Accordingly, a transient nucleos(t)ide-resistant HepG2 cell line carrying ETV resistance substitutions within reverse transcriptase (rt) (rtL180M/S202G/M204V) was established [33]. The expression of this virus and its loss of susceptibility to ETV were characterized. The viral replication EC_50_ of ETV was 6.1 nM in resistant cells, displaying a 1.9-fold shift in susceptibility to ETV compared with the wild-type virus (3.2 nM) (Figure S4A). However, the viral replication EC_50_ of C-39 (84 nM) observed in ETV-resistant cells was highly similar to the 78 nM EC_50_ in wild-type HBV expressing cells (Figure S4B), indicating that the nucleos(t)ide-resistant HBV variant remained sensitive to inhibition by C-39.

In vitro combination of CAMs with NAs showing additive effects in reducing both HBV DNA replication and the production of viral particles would support the premise of combination treatment with antiviral reagents with different mechanisms of action. Indeed, when HepAD38 cells were treated with 5 nM ETV alone or together with increasing concentrations of C-39 (0–10 μM), further inhibition of HBV DNA secretion and intracellular HBV DNA levels were observed in the combination group (Figure S4C,D).

### 3.7. C-39 Induced HBc Aggregation and Modulates Capsid Assembly

**Figure 7:**
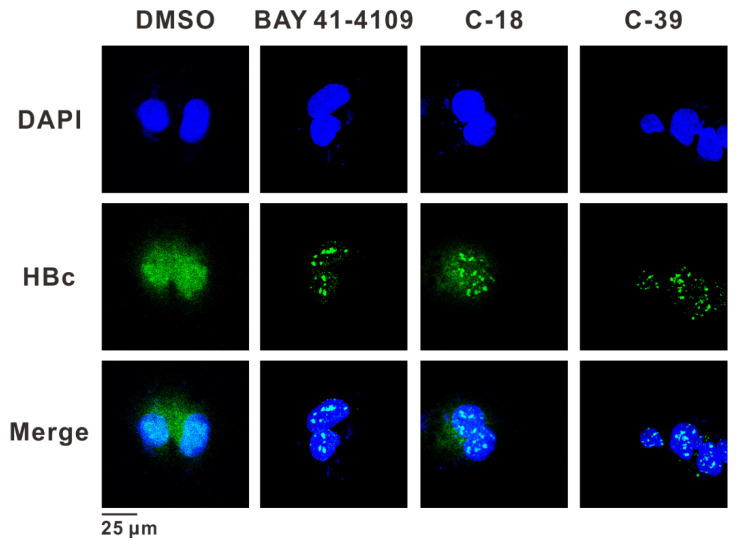
Effects of C-39 on the HBc aggregation. Confocal immunofluorescence microscopy images showing HBV core protein (in green) distribution in HepAD38 cells after 3 days of incubation of DMSO, BAY 41-4109 (2 μM), C-18 (5 μM), and C-39 (5 μM). Nuclei are DAPI stained (blue). Scale bar: 25 μm.

Immunofluorescence microscopy was used to investigate the effect of CAM treatment on the assembly and localization of Cp and capsid particles in HepAD38 cells. C-39, C-18, and BAY 41-4109 induced punctate HBc aggregation within the nucleus. C-39 and BAY 41-4109 also significantly decreased the HBc level (Figure 7), closely consistent with the above Western blotting assays. Transmission electron microscopy was used to intuitively show the capsid changes after the C-39 treatment. Recombinant Cp149 protein was purified in the form of a dimer and incubated in an assembly buffer with compounds for the indicated times. As shown in Figure S5, intact HBV core particles about 30 nm in diameter were observed in the DMSO control group (a,b). Compared with the large irregular particles induced by BAY 41-4109 (c,d), C-39 effectively accelerated the formation of capsid-like particles with time (e–h). Moreover, distorted and inflated particles were seen under high microscope magnification (f,h).

**Figure 8:**
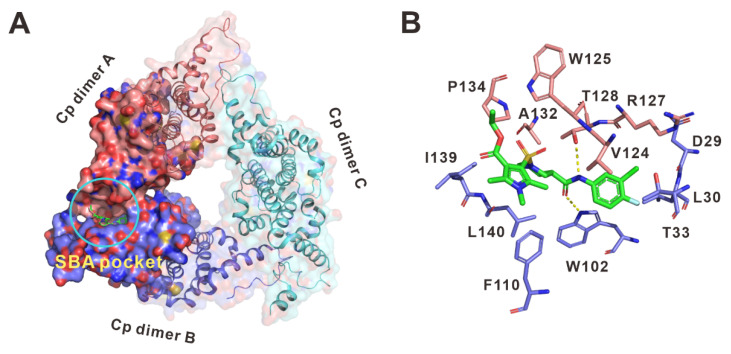
Putative binding mode of C-39 with HBV core protein. (**A**) Surface view of the putative binding model of C-39 in the SBA pocket. (**B**) Detailed molecular interactions of the HBV core proteins with C-39.

To gain insight into the interactions between C-39 and the core protein, we docked the C-39 to the SBA binding site. Molecular docking studies showed that the proposed binding mode of C-39 was like that of C-18. C-39 occupied a gap between the hydrophobic surfaces of the two adjacent Cp subunits (SBA pocket) (Figure 8A). The chloro-fluor-phenyl group was bound in a hydrophobic pocket surrounded by residues D29, L30, T33, W102, V124, R127, and T128. The nitrogen of the phenylcarboxamide moiety in C-39 formed a key hydrogen bond with Thr128 (dimer A), whereas the oxygen of this group formed an additional hydrogen bond with Trp102 (dimer B). The trimethyl pyrrole group pointed toward the solvent-exposed area and contacts with Phe110, Trp125, Pro134, and Ile139 (Figure 8B). These interactions could stabilize the Cp dimer–dimer complex.

## 4. Discussion

Current treatment of hepatitis B is mainly limited to NAs and peg-IFN-α, where a functional cure is rarely achieved. Therefore, it is of high interest to develop therapeutic agents with different MOAs (e.g., CAMs). HBV Cp is essential to many aspects of the HBV life cycle, including nucleocapsid assembly, viral pgRNA encapsidation, viral DNA replication, and cccDNA amplification [8]. These multiple effects make the core an attractive target for the development of new chronic hepatitis B (CHB) therapies. In this study, we explored the chemical space and identified potent CAM-based anti-HBV agents.

The initial screening campaign yielded 5 out of 24 compounds that exhibited >80% inhibition of extracellular HBV DNA, reflecting the high performance of our high throughput virtual screening-based strategy. Three new chemotypes, including triazoles (C-7), [1,2,4]triazolo[4,3-a]pyridines (C-16, C-25), and *N*-sulfonylpiperidine-3-carboxamides (C-18, C-39), were uncovered, thus providing new start points for the structural optimization. All these compounds together with certain reported CAMs, for example, NVR 3-778 [24], GLP-26 [27], HF9C6 [44], and oxadiazepinones [45], share a phenylcarboxamide moiety (ring A), which is supposed to bind in a hydrophobic region in the SBA binding site. However, the B rings are very different, indicating high group tolerance at this position. A similar characteristic was also seen in a series of phenylcarboxamide-based CAMs [46,47].

SAR exploration of SPCs demonstrated that groups toward the solvent region (R groups) fine-tune the anti-HBV activities. Surprisingly, SPCs behaved very differently in the capsid formation assay, although with potent HBV DNA suppressing activity. C-18 and C-39 dramatically perturbed HBc, encapsidated pgRNA, and capsid formation, whereas other SPCs did not. For example, C-19 had no significant impact on these Cp-associated molecules. The results demonstrated once again that slight changes in compounds with the same scaffold can result in different thermodynamic outcomes of capsid assembly, which is consistent with the different morphological consequences induced by two very similar SBA derivatives [38,48].

Both C-39 and ETV were able to effectively inhibit HBV DNA replication in the HepAD38 inducible system, transient HepG2-HBV1.3 cells, and HepG2-NTCP de novo infection model. In contrast to ETV treatment, C-39 also significantly decreased the levels of the biomarkers of cccDNA (HBeAg and 3.5 kb RNA) in the three culture models. This can be attributed to their distinct MOA. C-39 perturbs nucleocapsid formation and package of RNA, whereas ETV directly blocks the reverse transcription process, thus preventing the degradation of pgRNA. In the de novo infection model, C-39 prevented cccDNA establishment only in the early post-entry step. The possible underlying mechanism is that C-39 destroyed the integrity of capsid before the release of rcDNA into the nucleus. With low micromolar C-39 or BAY 41-4109 treatment, a strong decrease in 3.5 kb RNA was observed, which could be responsible for the decrease in the HBc. The reduction in HBeAg was most probably due to the decrease in intracellular RNA accumulation or a direct effect on HBeAg by CAMs, which could inhibit HBeAg secretion by inducing precore protein aggregation [28,49]. The reduction in the levels of intracellular pgRNA correlated well with the rapid decrease of intracellular HBV DNA, which can most likely be attributed to the fact that C-39 inhibits reverse transcription by interfering with the encapsulation of pgRNA into the capsid. Thus, intracellular HBV DNA levels in both cell lines were efficiently reduced after treatment with C-39. All the above suggested that C-39 targeted the intracellular HBV replication cycle.

Long-term treatment with NAs as antiviral drugs is prone to the selection of resistant HBV variants that carry mutations in the viral rt protein. The main HBV mutants (i.e., rtV173L, rtL180M, S202G, rtM204V, and rtN236T, single or in combination) resistant to NAs are susceptible to CAMs of HBV replication [33,50,51,52]. Because of its distinct MOA from the NA class of inhibitors, C-39 still maintained potency against the ETV-resistant HBV variant. Combination treatment with C-39 and ETV demonstrated their additive antiviral activity at increasing doses of CAM. It may, therefore, be beneficial to combine CAMs and NAs to increase antiviral replication suppression.

## 5. Conclusions

In this study, we presented the discovery of a new class of HBV CAM. SAR exploration of SPCs permitted the identification of C-39. Primary investigation revealed that C-39 interacts with Cp, thus interfering with the assembly of normal capsids and disrupting the HBV replication cycle. The discovery of C-39 provides a new research direction for the chemical structure of the next generation of anti-HBV CAMs.

## Figures and Tables

**Table 1:** The anti-HBV activity of C-7, C-9, C-16, C-18, C-19, ETV, and BAY 41-4109 in stable HBV-replicating HepAD38 cells.

Compounds	HepAD38		
	EC_50_ (μM)	CC_50_ (μM)	SI
C-7	1.83 ± 0.44	>100	>54.7
C-9	1.14 ± 0.23	26.6 ± 2.0	23.4
C-16	1.29 ± 0.35	72.4 ± 1.9	56.3
C-18	0.11 ± 0.03	14.8 ± 0.7	131.2
C-19	0.39 ± 0.13	75.3 ± 5.2	194.1
ETV	0.003 ± 0.001	ND	ND
BAY 41-4109	0.067 ± 0.01	30 ± 1.9	447

EC_50_, 50% effective concentration; CC_50_, 50% cytotoxic concentration; SI, selectivity index, SI = CC_50_/EC_50_; ND, not determined. All experiments were repeated at least three times.

**Table 2:** The anti-HBV activity of C-25–C-42 in HepAD38 or HepG2-HBV1.3 cells.

Compound	Structure	EC_50_ (μM) or Residual HBV DNA Level (%)	CC_50_ (μM)	SI
C-25	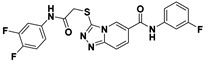	0.29 ± 0.02 ^a^	33.2 ± 1.9 ^a^	114.6 ^a^
C-26	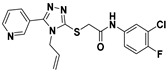	48.78 *	ND	ND
C-27	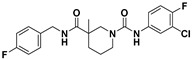	1.12 ± 0.05 ^a^	81.5 ± 5.8 ^a^	72.8 ^a^
C-28	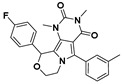	60.65 *	ND	ND
C-29	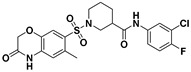	1.29 ± 0.01 ^a^	18.2 ± 2.2 ^a^	14.1 ^a^
C-30	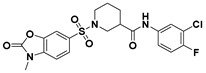	33.59 *	ND	ND
C-31	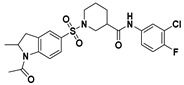	0.26 ± 0.03 ^a^	25.0 ± 3.3 ^a^	96.0 ^a^
C-32	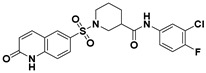	72.76 *	ND	ND
C-33	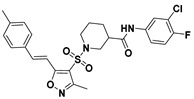	68.77 *	ND	ND
C-34	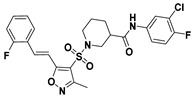	121.46 *	ND	ND
C-35	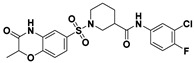	0.41 ± 0.01 ^a^	9.8 ± 1.0 ^a^	23.9 ^a^
C-36	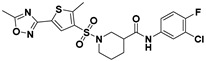	54.09 *	ND	ND
C-37	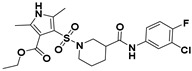	0.35 ± 0.05 ^a^	11.0 ± 0.7 ^a^	31.3 ^a^
C-38	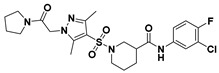	40.31 *	ND	ND
C-39	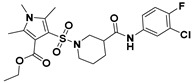	0.056 ± 0.013 ^a^	>100 ^a^	>1786 ^a^
		0.075 ± 0.016 ^b^	>100 ^b^	>1333 ^b^
C-40	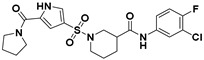	0.39 ± 0.01 ^a^	12.6 ± 0.7 ^a^	32.3 ^a^
C-41	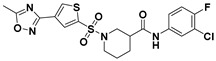	0.062 ± 0.013 ^a^	95.6 ± 15.7 ^a^	1542 ^a^
C-42	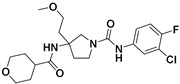	137.39 *	ND	ND

EC_50_, 50% effective concentration; CC_50_, 50% cytotoxic concentration; SI, selectivity index, SI = CC_50_/EC_50_. ^a^ EC_50_ or CC_50_ values determined in HepAD38 cells. ^b^ Values determined in HepG2-HBV1.3 cells. * Residual HBV DNA level at 20 μM compounds relative to DMSO control. ND, not determined. All experiments were repeated at least three times.

## Data Availability

The obtained and analyzed data of this study are available from the corresponding author on request.

## Supplementary Materials

The following supporting information can be downloaded at: https://www.mdpi.com/article/10.3390/v14020348/s1, Table S1: Primer sequences for RT-qPCR; Table S2: Chemical structures and anti-HBV DNA activities of C-1–C-24; Figure S1: C-18 inhibited HBV replication and capsid formation in HepG2-HBV1.3 cells; Figure S2: C-39 significantly inhibited HBV replication in HepG2-HBV1.3 cells. Figure S3: C-39 significantly inhibited HBV replication in the de novo infection model. Figure S4: C-39 inhibited the ETV-resistant HBV replication and the combination of C-39 and ETV enhanced the suppression of HBV replication. Figure S5: Electron microscopy analysis of the effects on capsid assembly. 1H NMR spectra of SPCs.
