# Supplementary material for: Structure-Based Discovery of N-Sulfonylpiperidine-3-Carboxamides as Novel Capsid Assembly Modulators for Potent Inhibition of HBV Replication
Source: Viruses. 2022 Feb 8;14(2):348. doi: 10.3390/v14020348 (PMC8876525; doi:10.3390/v14020348)
Supplement: Supplementary file 1 [file viruses-14-00348-s001.zip › viruses-1543846-supplementary.pdf]

viruses

MDPI

Supplementary Materials

Structure-Based Discovery of *N*-Sulfonylpiperidine-3-carboxamides as Novel Capsid Assembly Modulators for Potent Inhibition of HBV Replication

Yang Yang †, Yu Yan †, Jiaxin Yin, Jie Hu, Xuefei Cai, Jieli Hu, Jie Xia, Kai Wang, Ni Tang * and Luyi Huang *

Table S1. Primer sequences for RT-qPCR.

HBV 3.5 kb RNA (nt) 2150 to 2300	Former primer : 5′-CCTAGTAGTCAGTTATGTCAAC-3′
	Reverse primer: 5′-TCTATAAGCTGGAGGAGTGCGA-3′
Total HBV RNAs (nt) 3058 to 3158	Former primer : 5′- ACCGACCTTGAGGCATACTT-3′
	Reverse primer : 5′- GCCTACAGCCTCCTAGTACA -3′
HBV core DNA (nt) 1770 to 2105	Former primer : 5′-CCTCTTCATCCTGCTGCT-3′
	Reverse primer : 5′-AACTGAAAGCCAAACAGTG-3′
HBV cccDNA (nt) 1545 to 1899	Former primer : 5′-CTCCCCGTCTGTGCCTTCT-3′
	Reverse primer : 5′-CCCCAAAGCCACCCAAG-3′
	cccDNA probe: FAM-ACGTCGCATGGAGACCAC-CGTGAACGCC-TAMRA

Table S2. Chemical structures and anti-HBV DNA activities of C-1 – C-24.

Compound	Vendor ID	Structure	Residual HBV DNA Level (%) at 20 μM
C-1	STL173465	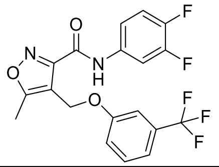	62
C-2	STL390663	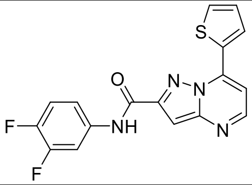	44
C-3	STL102917	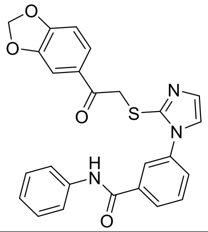	22

C-4	STK654654	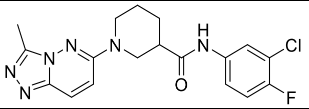	21
C-5	STL344232	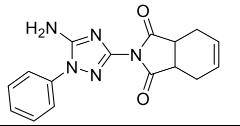	51
C-6	STK607709	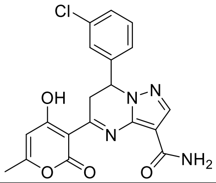	36
C-7	5236-0418	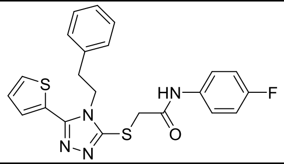	13
C-8	D359-0746	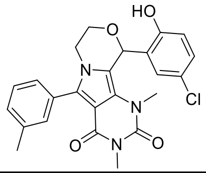	28
C-9	L949-2795	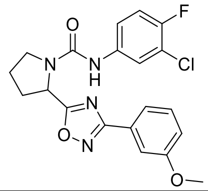	11
C-10	C453-0014	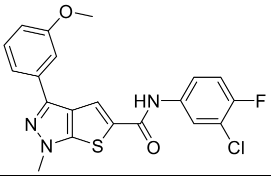	29
C-11	G214-4780	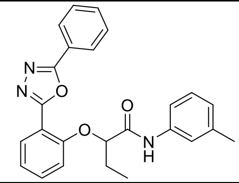	59
C-12	8018-8334	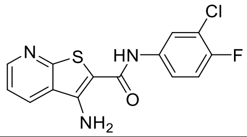	36
C-13	K889-0488	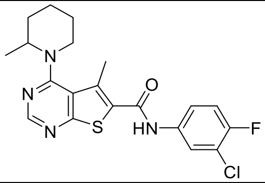	44
C-14	K838-0091	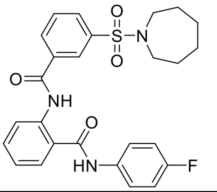	68

C-15	G808-0068	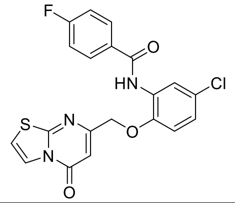	60
C-16	L860-0145	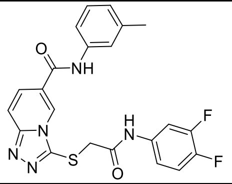	4
C-17	F550-3276	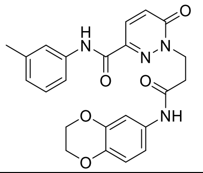	21
C-18	F184-1704	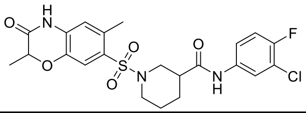	2
C-19	K781-8075	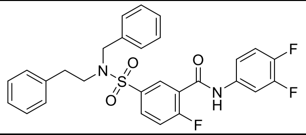	3
C-20	L810-0114	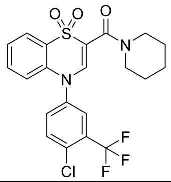	32
C-21	C326-0005	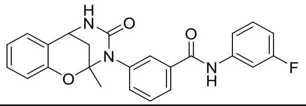	51
C-22	D473-1628	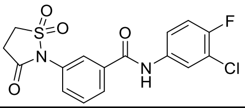	29
C-23	8017-8758	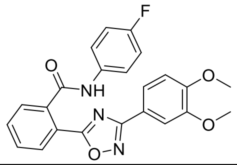	27
C-24	F589-0013	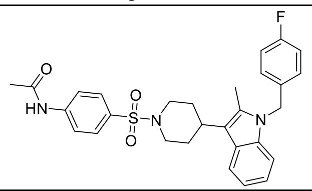	53

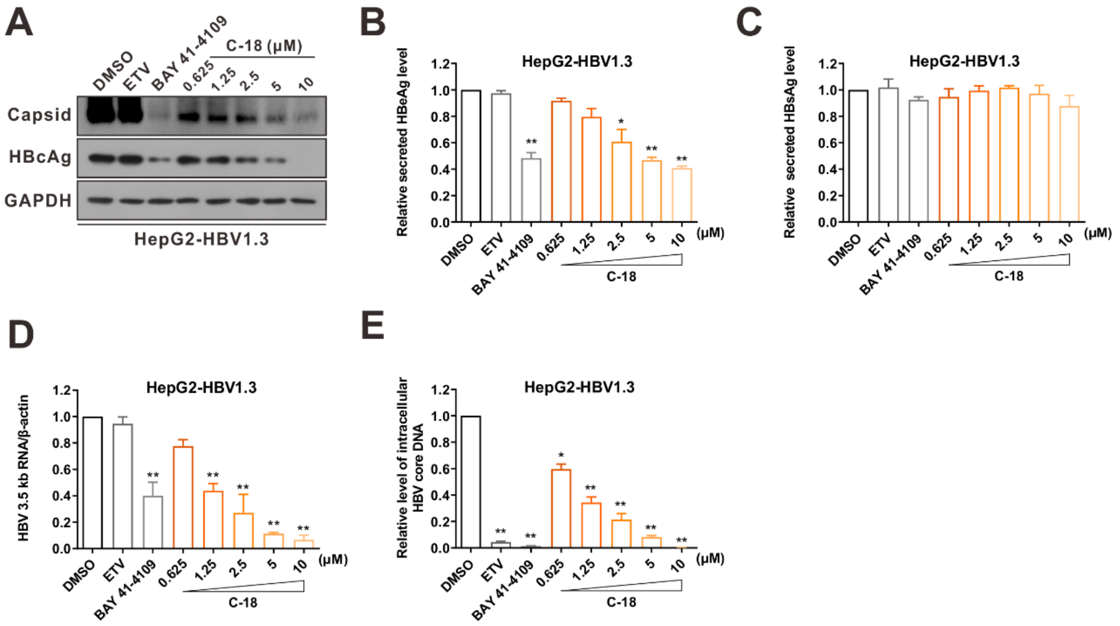

Figure S1. C-18 inhibits HBV replication and capsid formation in HepG2-HBV1.3 cells. (**A**) Effects of C-18 on intracellular HBV capsid formation and HBc. HepG2-HBV1.3 cells were incubated with indicated concentrations of C-18 for 6 days. Capsid and HBV core protein were probed with anti-HBV core antibody, and GAPDH was used as a loading control. (**B-E**) Effect of C-18 on HBV replication. HepG2-HBV1.3 cells were incubated with indicated concentrations of C-18 for 6 days. Secreted HBeAg (**B**) and HBsAg (**C**) were determined by ELISA, intracellular HBV 3.5 kb RNA (**D**) and HBV DNA (**E**) were measured by qPCR. ETV (25 nM), and BAY 41-4109 (2 μM) were used as controls. The data in (B-E) are representative of three independent experiments and are expressed as mean ± SD (Student's *t*-test was used to assess the statistical significance between DMSO group against any other treatment, *$p < 0.05$; **$p < 0.01$).

.

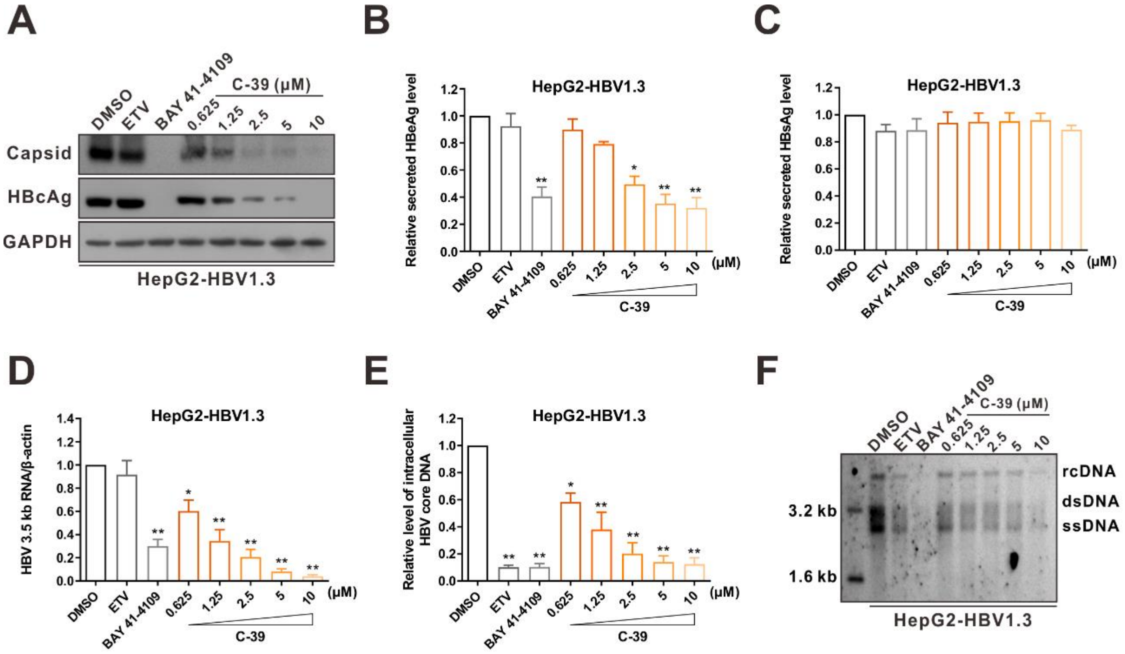

.

Figure S2. C-39 significantly inhibits HBV replication in HepG2-HBV1.3 cells. HepG2-HBV1.3 cells were incubated with the indicated concentrations of C-39 for 6 days. (**A**) Effects of C-39 on intracellular HBV capsid formation and HBc. HBV capsid and core protein were assessed by anti-HBV core antibody. (**B-F**) Effects of C-39 on HBV replication. Secreted HBeAg (**B**) and HBsAg (**C**) were determined by ELISA, intracellular HBV 3.5 kb RNA (**D**) and core DNA (**E**) were measured by qPCR, and HBV replication intermediates (RIs) were assessed by Southern blotting assay (**F**). ETV (25 nM) and BAY 41-4109 (2 μM) were used as controls. The data in (**B-E**) are representative of three independent experiments and are expressed as mean ± SD (Student's *t*-test was used to assess the statistical significance between DMSO group against any other treatment, *$p < 0.05$; **$p < 0.01$).

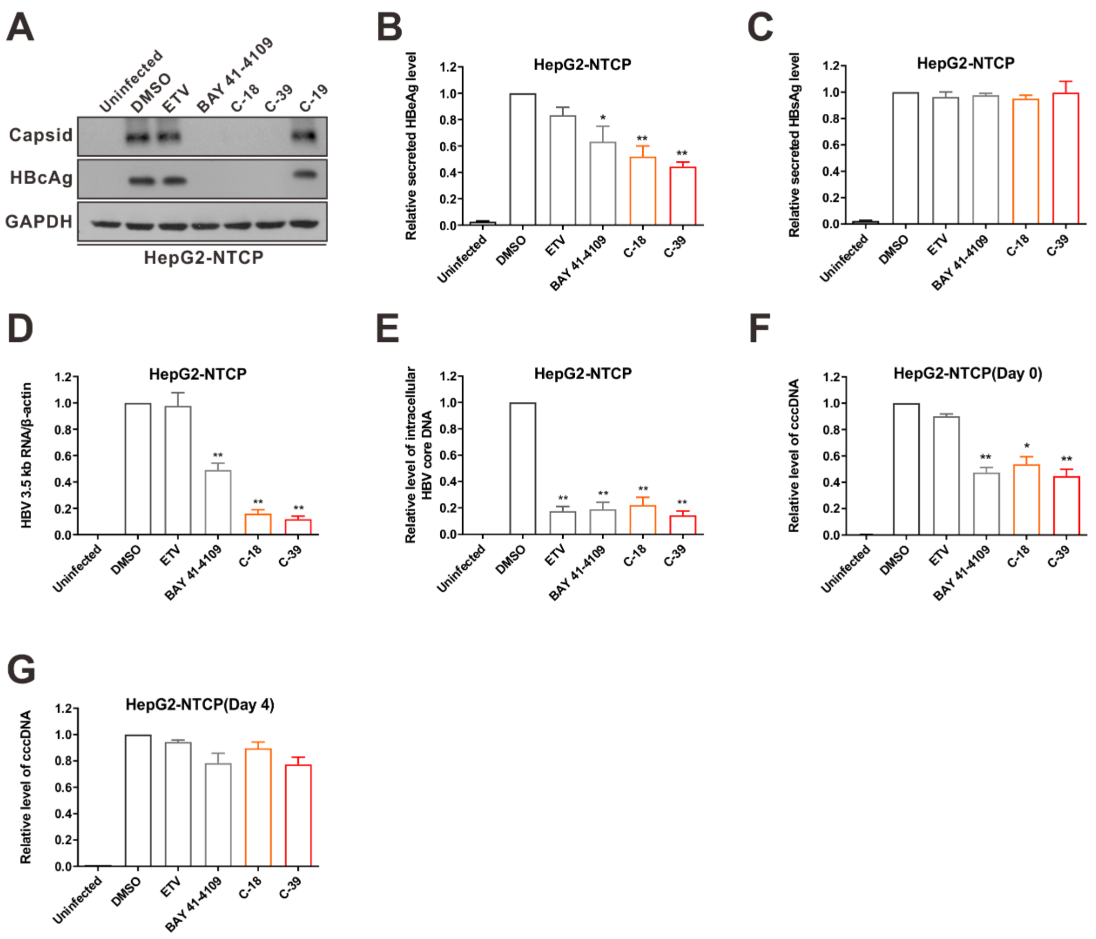

Figure S3. C-39 significantly inhibits HBV replication in de novo infection model. HepG2-NTCP cells were incubated with the indicated concentrations of C-39 for 6 days. (**A**) Effects of C-39 on intracellular HBV capsid assembly. HBV capsids and core protein were assessed by anti-HBV core antibody. (**B-F**) Effects of C-39 on HBV replication. Secreted HBeAg (**B**) and HBsAg (**C**) were determined by ELISA, intracellular HBV 3.5 kb RNA (D) and core DNA (**E**) were measured by qPCR. ETV (25 nM) and BAY 41-4109 (2 μM) were used as controls. (**F-G**) HBV cccDNA levels in HepG2-NTCP cells when HepG2-NTCP cells were infected with HBV and treated with different compounds either together with the viral inoculum (day 0, F) or at 4 days after infection (day 4, **G**). C-18, C-19, and C-39 were used at 5 μM. ETV (25 nM) and BAY 41-4109 (2 μM) were used as controls. The data in (**B-G**) are representative of three independent experiments and are expressed as mean ± SD (Student's *t*-test was used to assess the statistical significance between DMSO group against any other treatment, *$p < 0.05$; **$p < 0.01$).

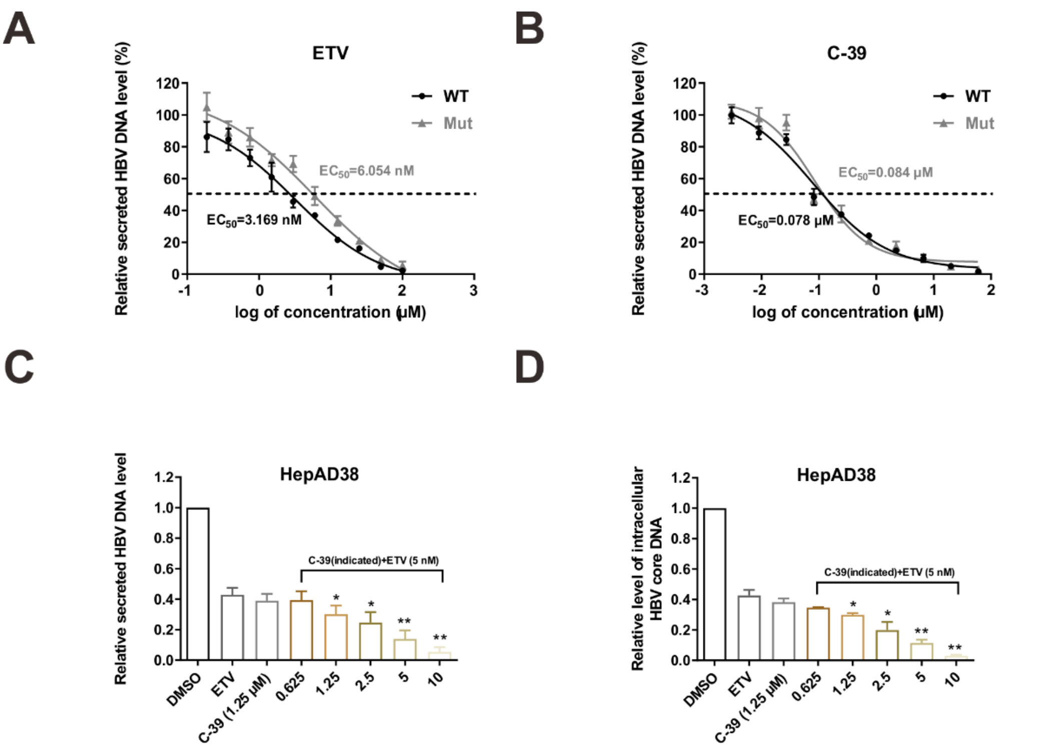

Figure S4. C-39 inhibits ETV-resistant HBV replication and the combination of C-39 and ETV enhances the suppression of HBV replication. Antiviral activity of ETV (**A**) and C-39 (**B**) by presence or absence of nucleos(t)ide-resistant mutations. Secreated HBV DNA (**C**) and intracellular HBV core DNA(**D**) levels in response to the treatment of ETV in combination with increasing doses of C-39. The data are representative of three independent experiments and are expressed as mean ± SD (Student's *t*-test was used to assess the statistical significance between combination against any other treatment, $*p < 0.05$; $**p < 0.01$).

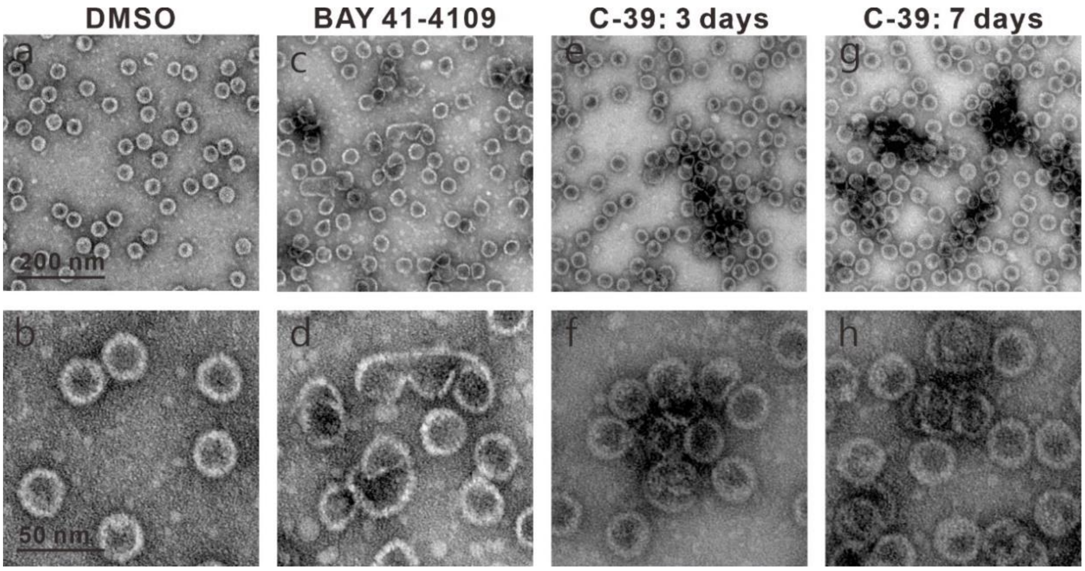

Figure S5. Electron microscopy analysis of the effects on capsid assembly**.** Recombinant Cp149 was treated with DMSO, 5 μM BAY 41-4109 for 3 days or 20 μM C-39 for 3 and 7 days in 300 mM NaCl and 150 mM HEPES (pH 7.5). Electron microscopic images of capsids are shown. Scale bars = 200 nm (up) and 50 nm (down).

^{1}H NMR Spectra of SPCs

C-18

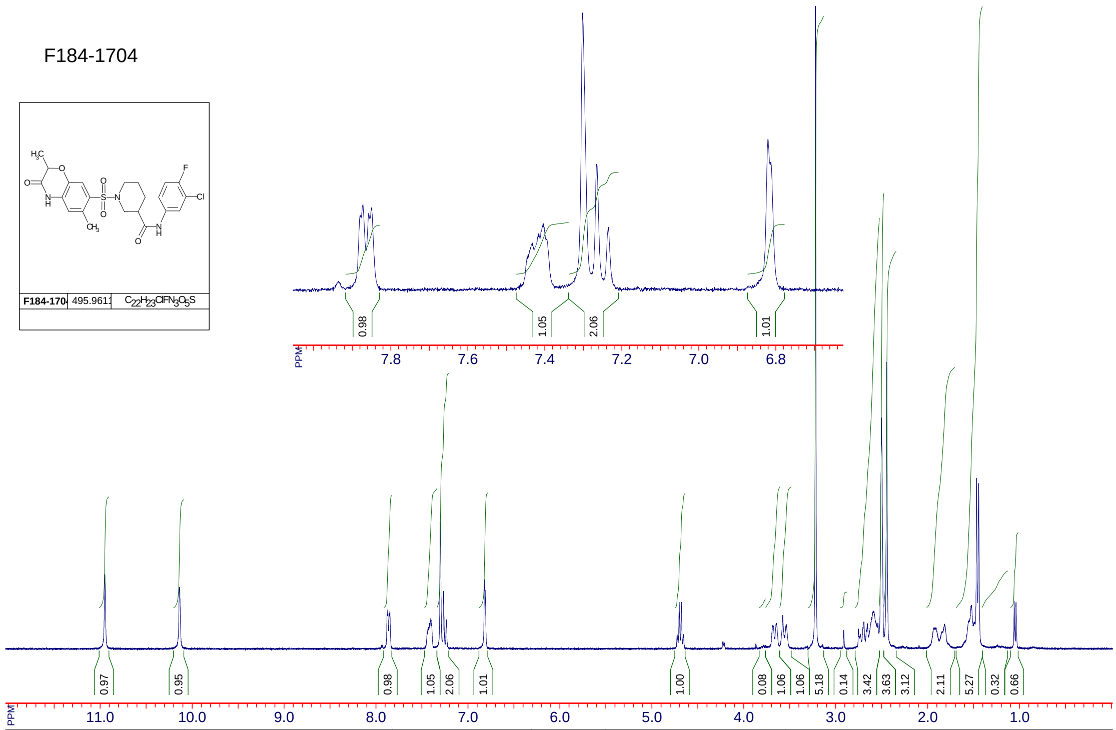

File name: F184-1704	Operator: MEL , Proc: OLE	SF: 300.1300 MHz	NSC: 1	PW: 0.00 usec,	Grade: OK(10); Expert: ;
DPX-300., 13-May-2006	Solvent:DMSO	SW: 5995 Hz	TE: 303 K	AQ: 3.00 sec, RD: 3 sec	Parameter file, XWIN-NMR == A12181_p5

C-29

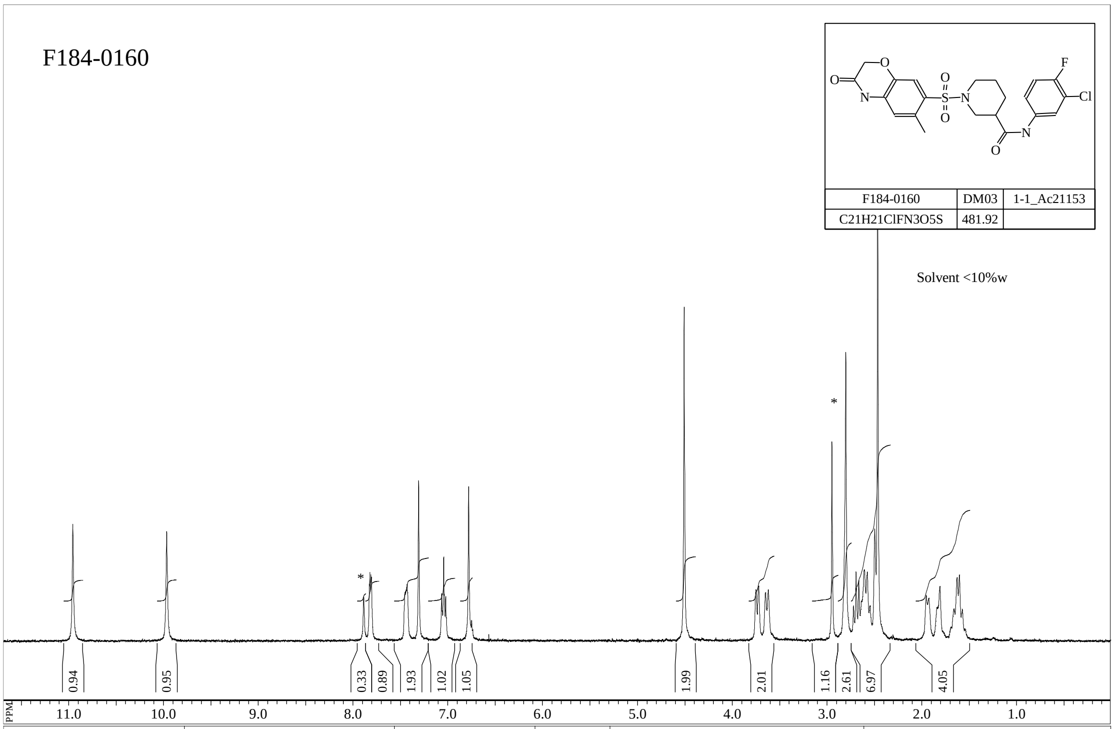

File name: F184-0160	Operator: NVV Expert: NVV	SF: 399.9525 MHz	NSC: 8	PW: 8.00 usec, RG: 14, SI: 16384	Grade: OK(10)
Date: 30-Mar-2006	Solvent: DMSO + CCl4	SW: 7502 Hz	TE: 300 K	AQ: 1.09 sec, RD: 1.50 sec	* F184- 0160. 30- Mar - 2006. 10*

C-30

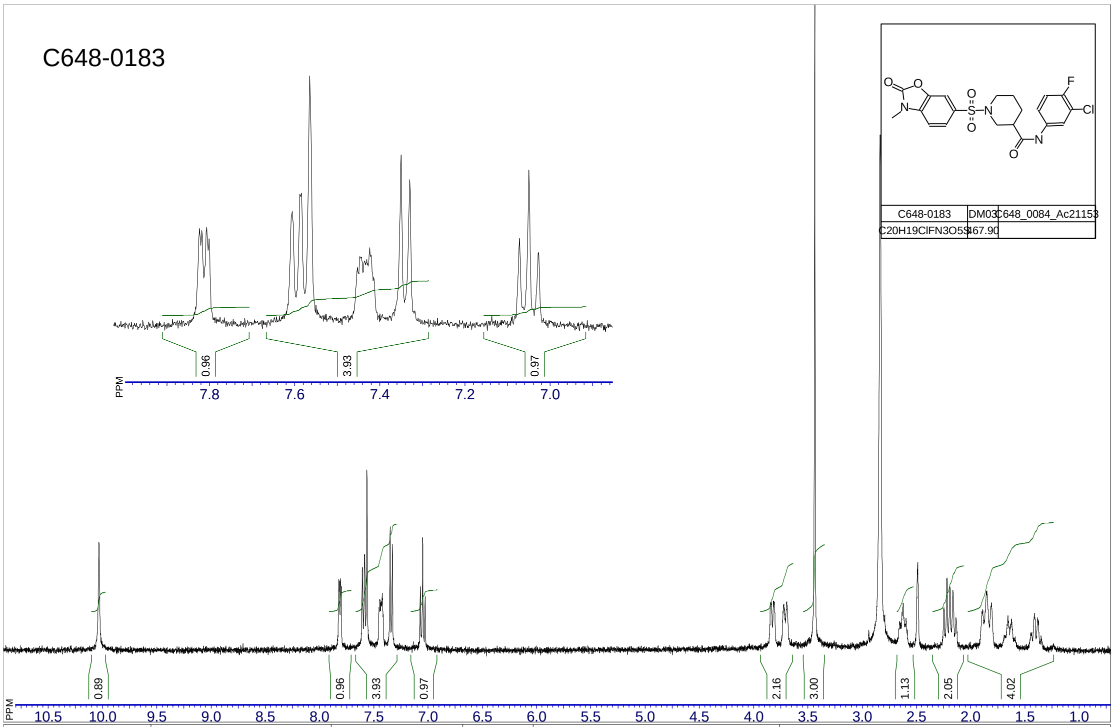

**File name: C648-0183**	Expert: BLJ	SF: 399.9525 MHz	NSC: 12	PW: 12.00 usec, RG: 28, SI: 16384	**Grade: OK(00)**
Date: 11-Jun-2003	Solvent: DMSO-d6+CCL4	SW: 7502 Hz	TE: 300 K	AQ: 1.09 sec, RD: 1,5 sec	*C648-0183. 11-Jun-2003. 00*

C-31

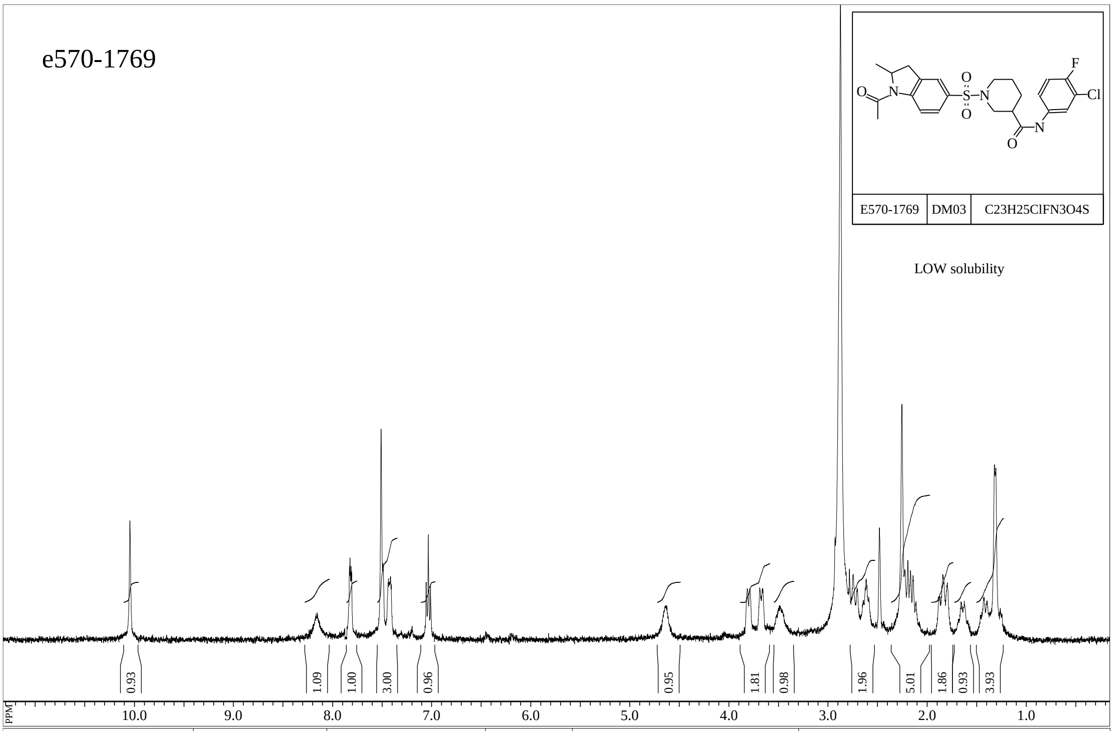

**File name: e570-1769**	Operator: VVN	SF: 399.9525 MHz	NSC: 12	PW: 12.00 usec, RG: 28	**Grade: OK(00)**
Date: 01-May-2004	Solvent: DMSO	SW: 7502 Hz	TE: 300 K	AQ: 1.09 sec, RD: 1.50 sec	*e570-1769. 01-May-2004. 00*

C-32

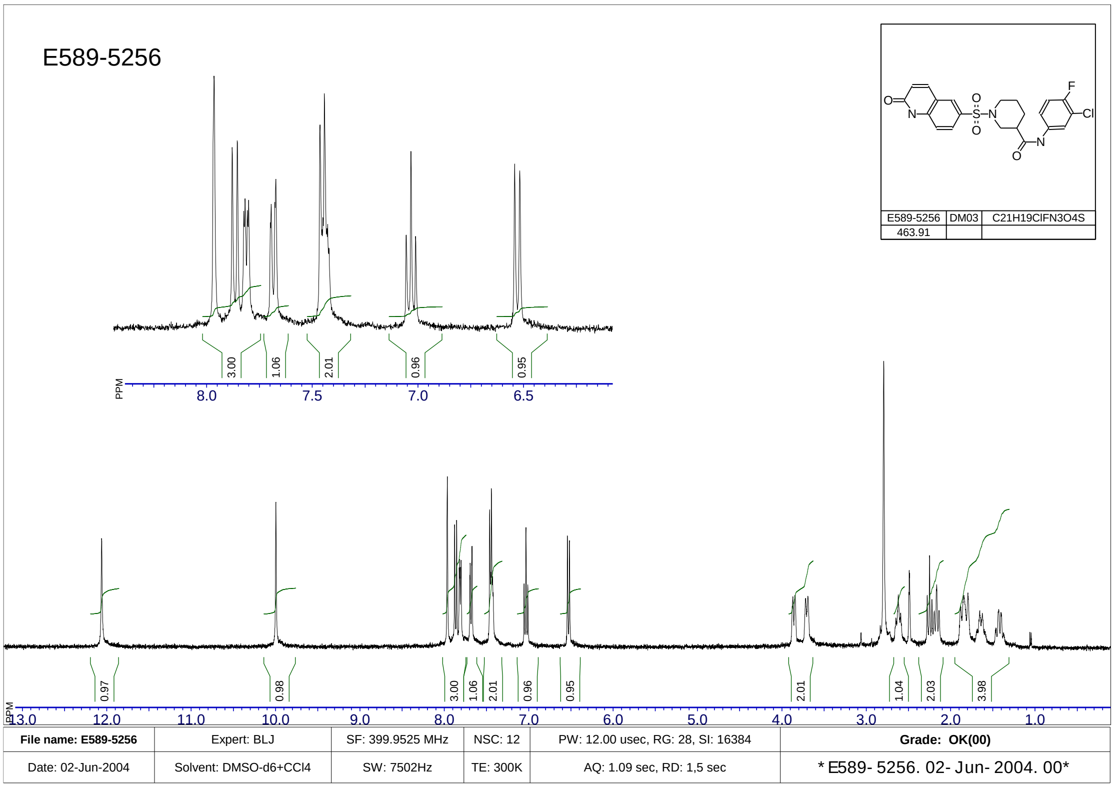

E589-5256
E589-5256 DM03 C21H19ClFN3O4S
463.91
3.00 1.06 2.01 0.96 0.95
0.97 0.98 3.00 1.06 2.01 0.96 0.95 2.01 1.04 2.03 3.98
PPM
File name: E589-5256
Expert: BLJ
SF: 399.9525 MHz
NSC: 12
PW: 12.00 usec, RG: 28, SI: 16384
Grade: OK(00)
Date: 02-Jun-2004
Solvent: DMSO-d6+CCl4
SW: 7502Hz
TE: 300K
AQ: 1.09 sec, RD: 1,5 sec
* E589- 5256. 02- Jun- 2004. 00*

C-33

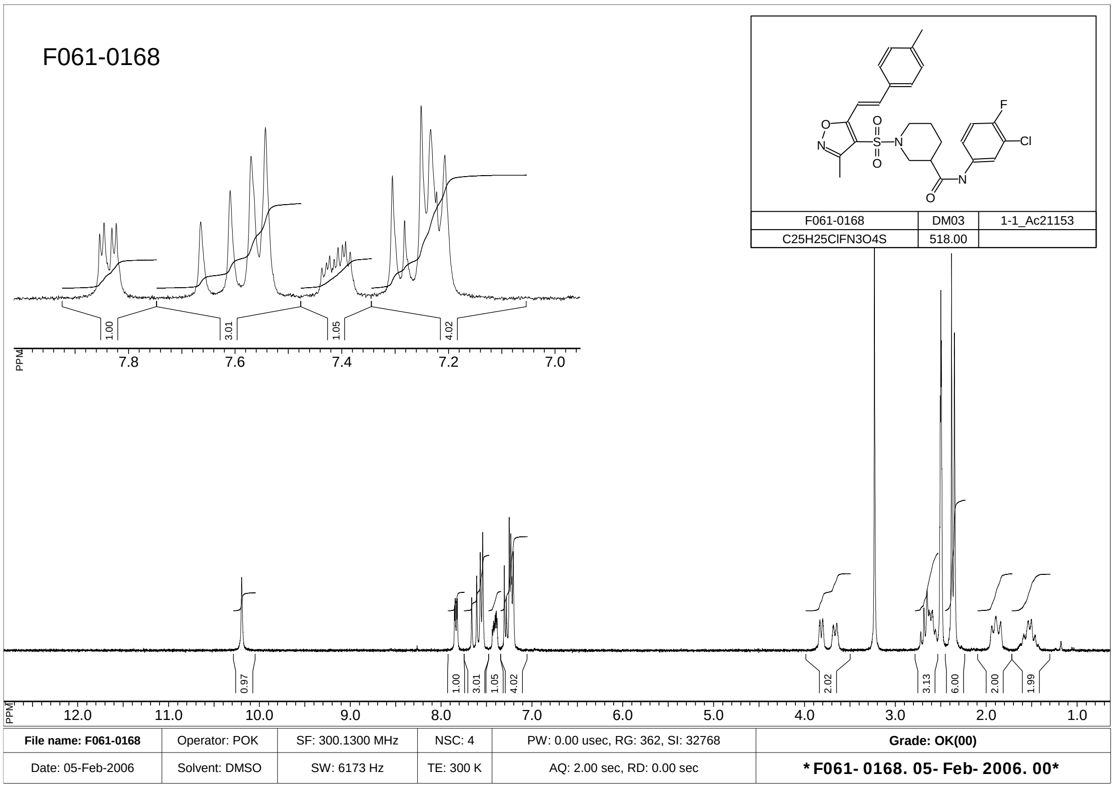

F061-0168
F061-0168 DM03 1-1_Ac21153
C25H25ClFN3O4S 518.00
1.00 3.01 1.05 4.02
0.97 1.00 3.01 1.05 4.02 2.02 3.13 6.00 2.00 1.99
PPM
File name: F061-0168
Operator: POK
SF: 300.1300 MHz
NSC: 4
PW: 0.00 usec, RG: 362, SI: 32768
Grade: OK(00)
Date: 05-Feb-2006
Solvent: DMSO
SW: 6173 Hz
TE: 300 K
AQ: 2.00 sec, RD: 0.00 sec
* F061- 0168. 05- Feb- 2006. 00*

C-34

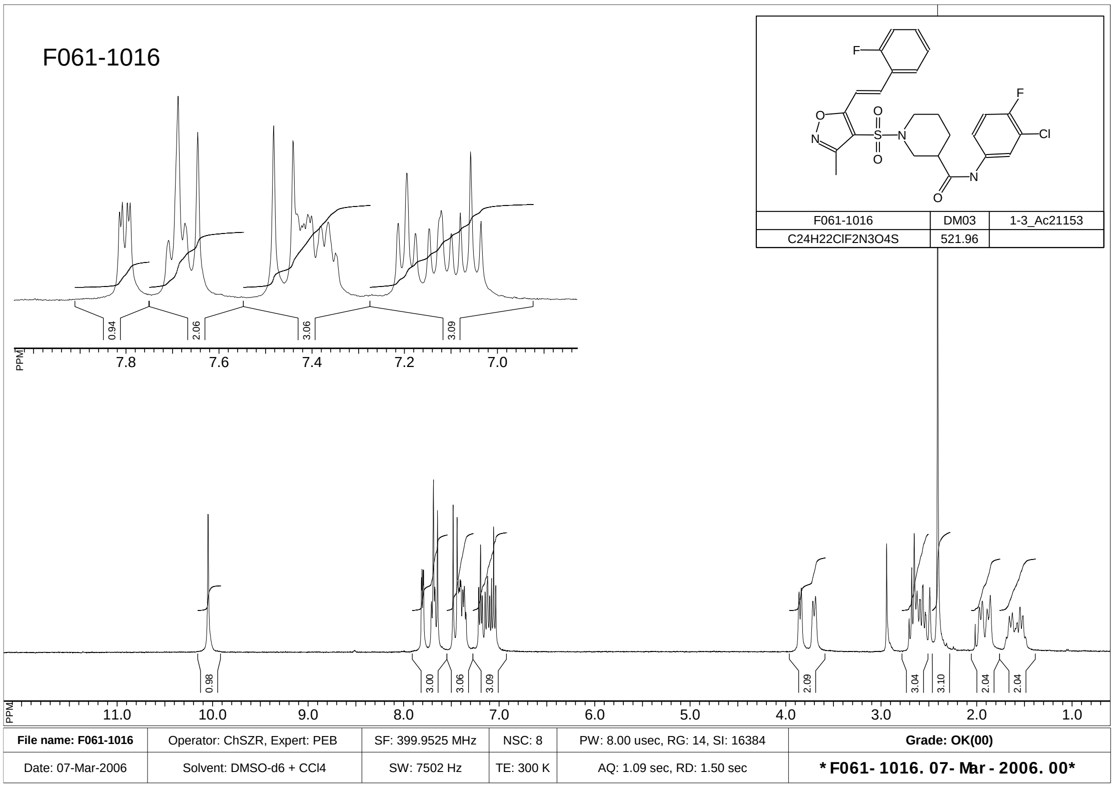
F061-1016
F061-1016 | DM03 | 1-3_Ac21153
C24H22ClF2N3O4S | 521.96
File name: F061-1016 | Operator: ChSZR, Expert: PEB | SF: 399.9525 MHz | NSC: 8 | PW: 8.00 usec, RG: 14, SI: 16384 | Grade: OK(00)
Date: 07-Mar-2006 | Solvent: DMSO-d6 + CCl4 | SW: 7502 Hz | TE: 300 K | AQ: 1.09 sec, RD: 1.50 sec | * F061- 1016. 07- Mar - 2006. 00*

C-35

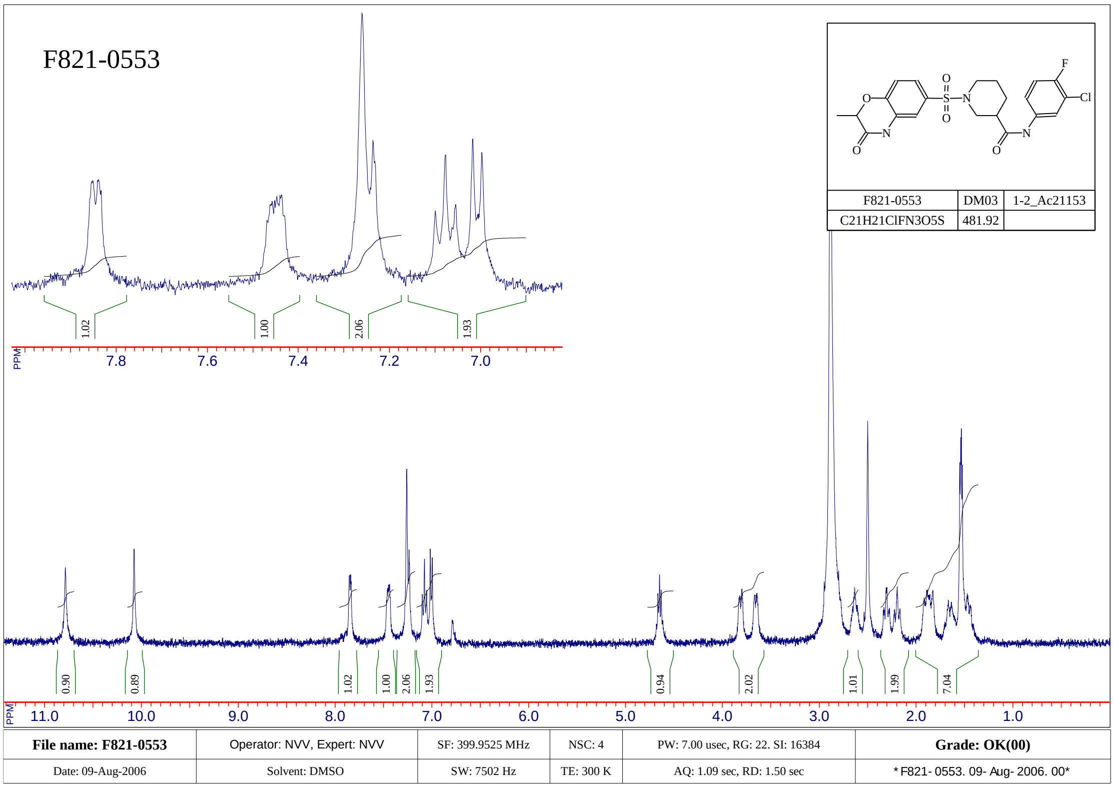
F821-0553
F821-0553 | DM03 | 1-2_Ac21153
C21H21ClFN3O5S | 481.92
File name: F821-0553 | Operator: NVV, Expert: NVV | SF: 399.9525 MHz | NSC: 4 | PW: 7.00 usec, RG: 22. SI: 16384 | Grade: OK(00)
Date: 09-Aug-2006 | Solvent: DMSO | SW: 7502 Hz | TE: 300 K | AQ: 1.09 sec, RD: 1.50 sec | * F821- 0553. 09- Aug- 2006. 00*

C-36

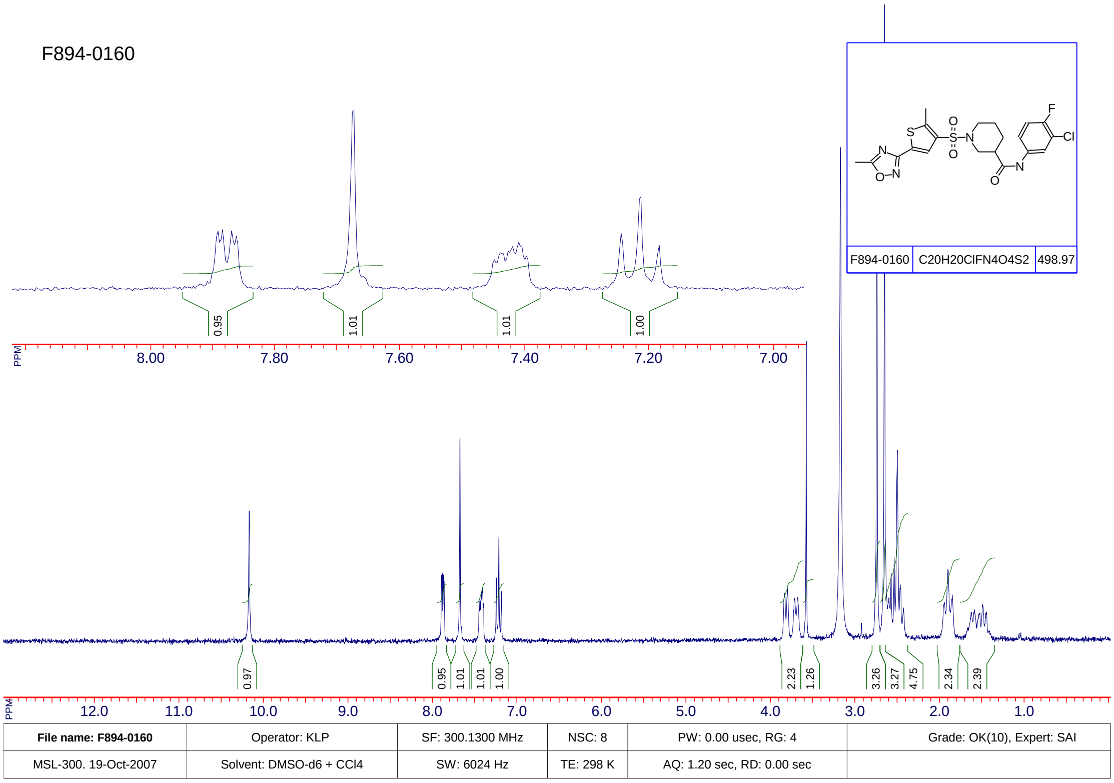
F894-0160
F894-0160
C20H20ClFN4O4S2
498.97
File name: F894-0160
Operator: KLP
SF: 300.1300 MHz
NSC: 8
PW: 0.00 usec, RG: 4
Grade: OK(10), Expert: SAI
MSL-300. 19-Oct-2007
Solvent: DMSO-d6 + CCl4
SW: 6024 Hz
TE: 298 K
AQ: 1.20 sec, RD: 0.00 sec

C-37

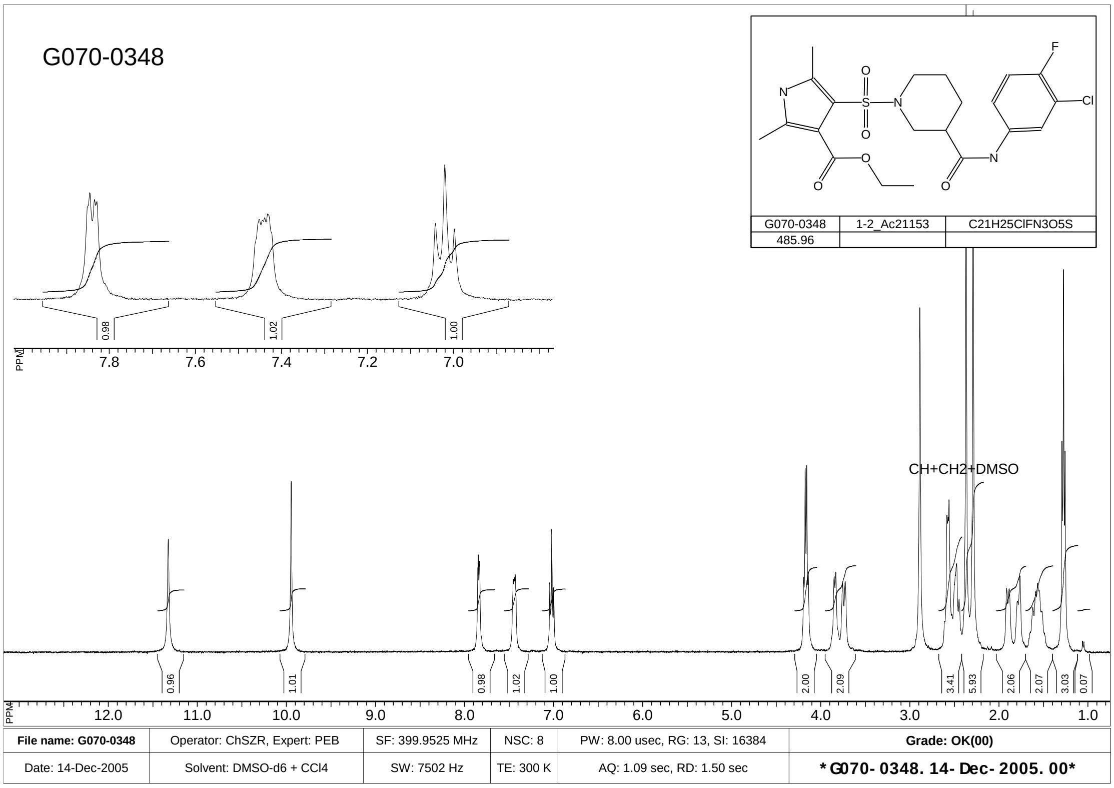
G070-0348
G070-0348
1-2_Ac21153
C21H25ClFN3O5S
485.96
CH+CH2+DMSO
File name: G070-0348
Operator: ChSZR, Expert: PEB
SF: 399.9525 MHz
NSC: 8
PW: 8.00 usec, RG: 13, SI: 16384
Grade: OK(00)
Date: 14-Dec-2005
Solvent: DMSO-d6 + CCl4
SW: 7502 Hz
TE: 300 K
AQ: 1.09 sec, RD: 1.50 sec
* G070- 0348. 14- Dec- 2005. 00*

C-38

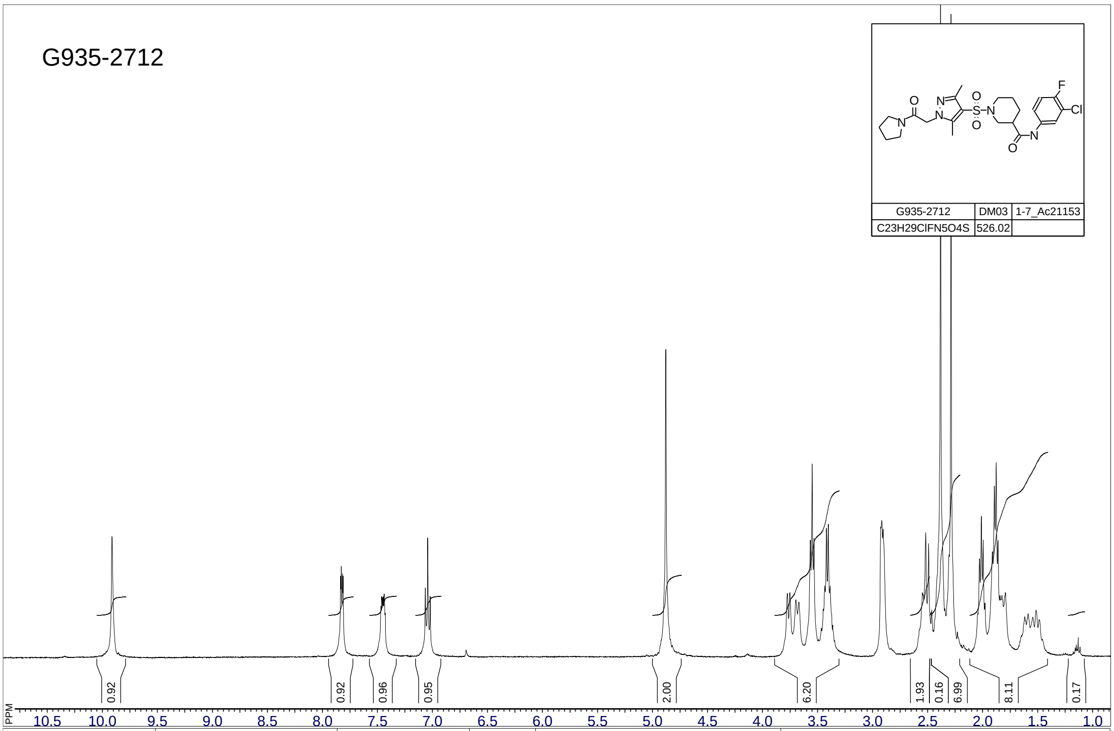

**File name: G935-2712**	Expert: BLJ	SF: 399.9525 MHz	NS: 8	PW: 12.00usec, RG: 14, SI: 16384	**Grade: OK(00)**
Date: 12-Jul-2006	Solvent: DMSO-d6+CCL4	SW: 7502Hz	TE: 300K	AQ: 1.09 sec, RD: 1,5sec	*G935-2712.12-Jul-2006.00*

C-39

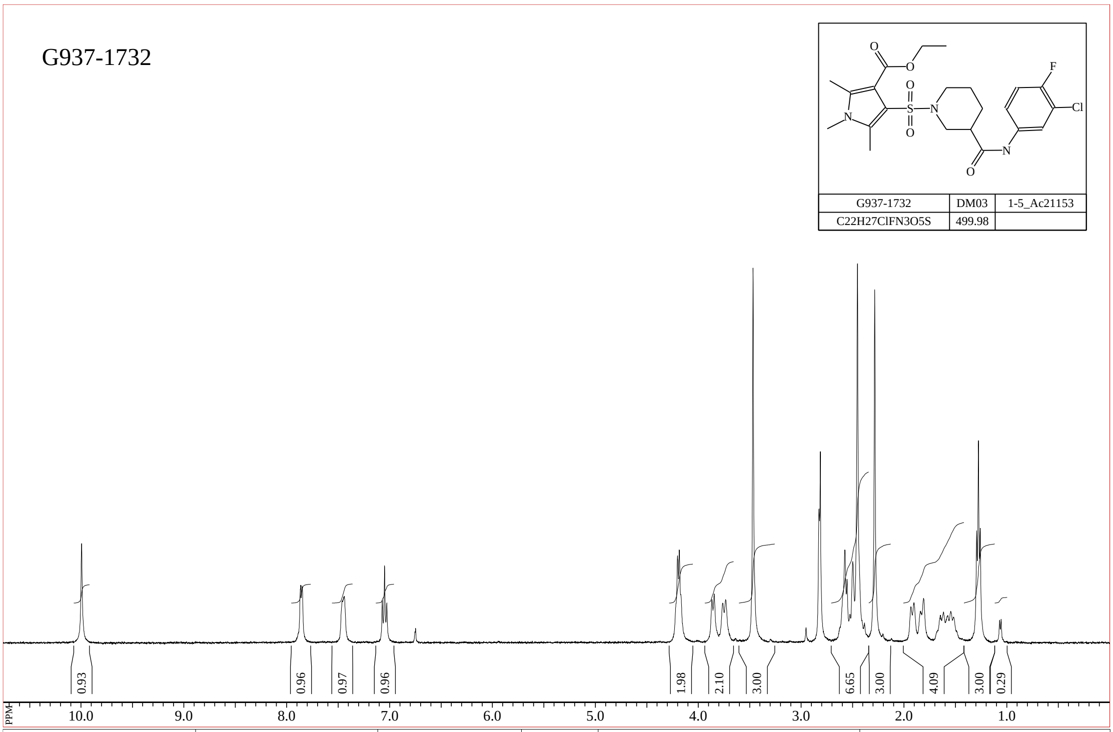

**File name: G937-1732**	Operator: , Expert: NVV	SF: 399.9525 MHz	NSC: 3	PW: 8.00 usec, RG: 14, SI: 16384	**Grade: OK(00)**
Date: 15-Dec-2005	Solvent: DMSO + CCl4	SW: 7502 Hz	TE: 300 K	AQ: 1.09 sec, RD: 1.50 sec	*G937-1732.15-Dec-2005.00*

C-40

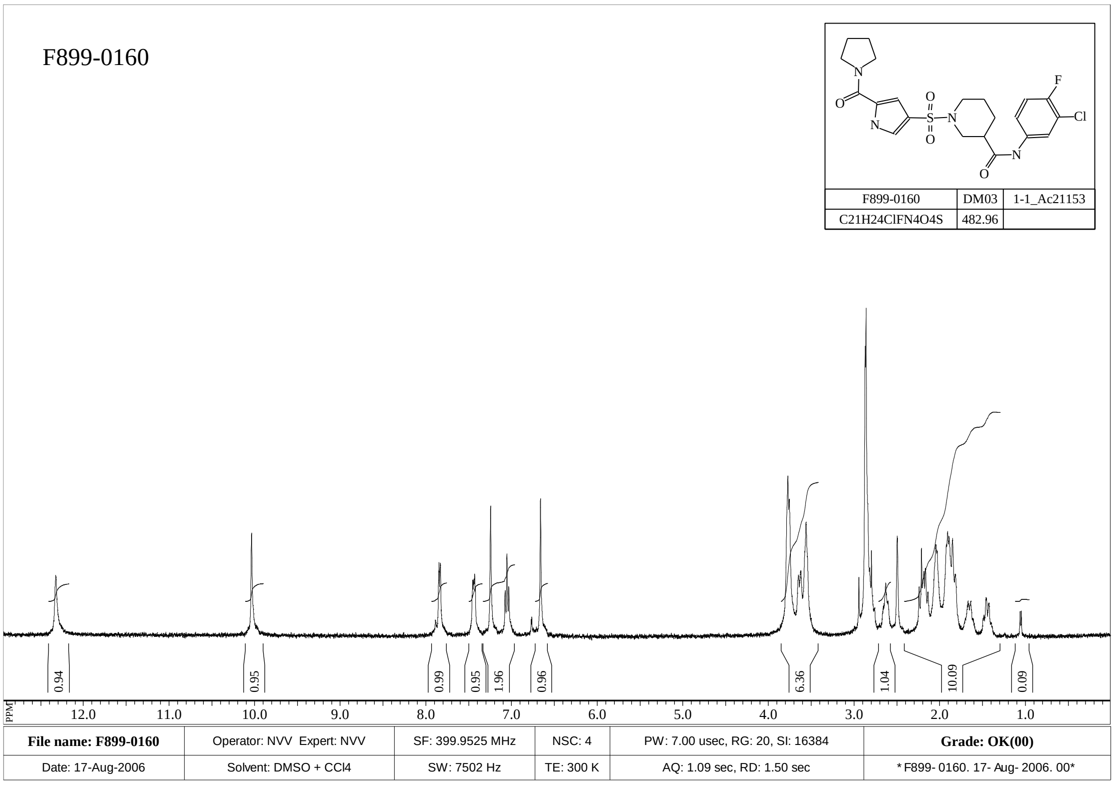

F899-0160
F899-0160 DM03 1-1_Ac21153
C21H24ClFN4O4S 482.96
File name: F899-0160
Operator: NVV Expert: NVV
SF: 399.9525 MHz
NSC: 4
PW: 7.00 usec, RG: 20, SI: 16384
Grade: OK(00)
Date: 17-Aug-2006
Solvent: DMSO + CCl4
SW: 7502 Hz
TE: 300 K
AQ: 1.09 sec, RD: 1.50 sec
F899- 0160. 17- Aug- 2006. 00

C-41

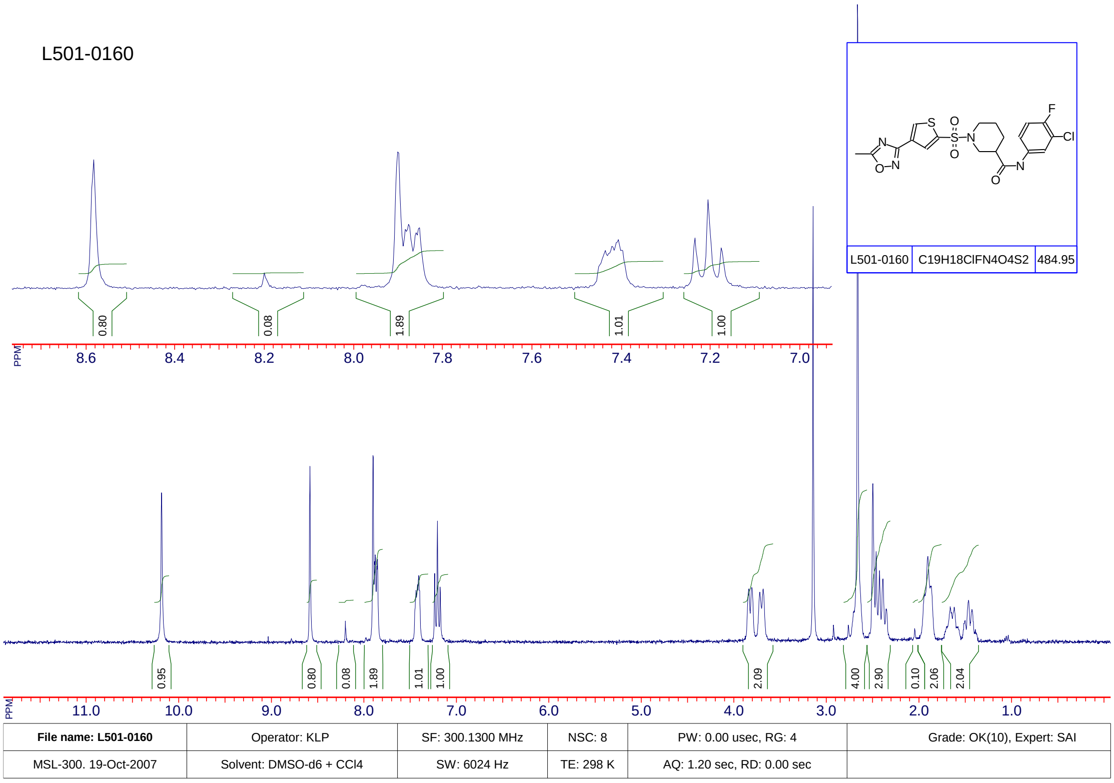

L501-0160
L501-0160 C19H18ClFN4O4S2 484.95
File name: L501-0160
Operator: KLP
SF: 300.1300 MHz
NSC: 8
PW: 0.00 usec, RG: 4
Grade: OK(10), Expert: SAI
MSL-300. 19-Oct-2007
Solvent: DMSO-d6 + CCl4
SW: 6024 Hz
TE: 298 K
AQ: 1.20 sec, RD: 0.00 sec
